# Human amniotic fluid contaminants alter thyroid hormone signalling and early brain development in Xenopus embryos

**DOI:** 10.1038/srep43786

**Published:** 2017-03-07

**Authors:** Jean-Baptiste Fini, Bilal B. Mughal, Sébastien Le Mével, Michelle Leemans, Mélodie Lettmann, Petra Spirhanzlova, Pierre Affaticati, Arnim Jenett, Barbara A. Demeneix

**Affiliations:** 1 UMR CNRS 7221, Evolution des Régulations Endocriniennes, Muséum National d’Histoire Naturelle, Sorbonne Université, 75231 Paris, France; 2 UMR CNRS TEFOR, Tefor Core Facility Paris-Saclay Institute of Neuroscience UMR 9197, CNRS, Université Paris-Saclay, France

## Abstract

Thyroid hormones are essential for normal brain development in vertebrates. In humans, abnormal maternal thyroid hormone levels during early pregnancy are associated with decreased offspring IQ and modified brain structure. As numerous environmental chemicals disrupt thyroid hormone signalling, we questioned whether exposure to ubiquitous chemicals affects thyroid hormone responses during early neurogenesis. We established a mixture of 15 common chemicals at concentrations reported in human amniotic fluid. An *in vivo* larval reporter (GFP) assay served to determine integrated thyroid hormone transcriptional responses. Dose-dependent effects of short-term (72 h) exposure to single chemicals and the mixture were found. qPCR on dissected brains showed significant changes in thyroid hormone-related genes including receptors, deiodinases and neural differentiation markers. Further, exposure to mixture also modified neural proliferation as well as neuron and oligodendrocyte size. Finally, exposed tadpoles showed behavioural responses with dose-dependent reductions in mobility. In conclusion, exposure to a mixture of ubiquitous chemicals at concentrations found in human amniotic fluid affect thyroid hormone-dependent transcription, gene expression, brain development and behaviour in early embryogenesis. As thyroid hormone signalling is strongly conserved across vertebrates the results suggest that ubiquitous chemical mixtures could be exerting adverse effects on foetal human brain development.

Brain development in all vertebrates requires thyroid hormones^1^,^2^. Severe thyroid hormone deficiency induces cretinism^3^. Recently, slightly lower or higher maternal thyroid hormone levels during early pregnancy were shown to be associated with decreased IQ and modified brain structure in children^4^. These data underline the previously underestimated role of thyroid hormones in early brain development^5^ and complement the well-established role for the hormone in later stages of brain development and maturation^1^.

Numerous studies have documented significant contamination of human populations and wildlife by multiple anthropogenic chemicals^6^,^7^. On average, over 30 anthropogenic chemicals are present in all American women, with 15 being ubiquitous, including in pregnant women^6^. Many of these chemicals are demonstrated or suspected thyroid hormone disruptors^8^,^9^, raising the question of whether current exposure to ubiquitous chemicals affects thyroid signalling and thereby early brain development. Even though certain xenobiotics have been investigated for their individual actions on specific endocrine axes, few studies have addressed their combined, or ‘cocktail’ effects. This lack of experimental data is striking given the increasing evidence that combinations of substances that individually have no adverse effect but can produce significant effects when tested as a mixture^10^,^11^.

To address how embryonic thyroid hormone signalling is affected by these 15 common chemicals, individually and in combination, we exploited the fluorescent *X. laevis* embryonic thyroid hormone reporter assay (XETA)^12^. This assay uses a transgenic line of *Xenopus laevis, Tg(thibz:eGFP),* which expresses GFP under the control of a 850 bp regulatory region of the TH/bZIP, a leucine zipper transcription factor highly sensitive to thyroid hormone regulation^13^,^14^. Using free-living tadpoles takes advantage of the high conservation of thyroid signalling across vertebrates, while providing access to early organogenesis, a developmental stage that is intractable for screening purposes in mammalian models. The XETA GFP readout informs on thyroid hormone disruption, with both increased and decreased fluorescence indicating altered hormone bioavailability.

Eleven of the 15 chemicals tested individually, exerted inhibitory or activating effects on thyroid hormone bioavailability in XETA. As synergistic effects of chemical mixtures without individual effects have been reported^10^,^15^, we established a mixture of the 15 ubiquitous chemicals at concentrations reported in human amniotic fluids (Table S1). We used this mixture at three different concentrations, where 1x represents the concentrations of individual chemicals reported in human amniotic fluid. Effects of exposure were determined on thyroid hormone bioavailability (XETA), brain gene expression and structure, and behaviour. Significant and dose-dependent effects were found in all assays, raising the question of potential adverse effects of current chemical exposures on foetal brain development.

## Results

In this work we analysed the consequences of human amniotic fluid contaminant exposure during embryonic development on thyroid hormone signalling and brain development. We first tested the thyroid hormone disruptive capacity of chemicals, individually and as a mixture, using a validated assay, the XETA^12^. Following the XETA, effects of chemical exposure were analysed on brain gene expression, neural proliferation, neuron and oligodendrocyte number and volume, and swimming behaviour.

### Eleven chemical contaminants of amniotic fluid disrupt thyroid hormone signalling

**Figure 1:**
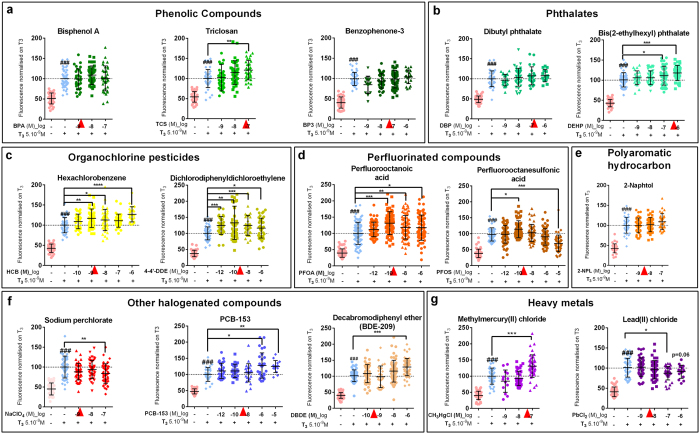
Thyroid disrupting activity of individual chemicals assessed with XETA. Screening of thyroid disrupting activity of molecules measured in humans with the Xenopus Embryonic Thyroid Assay (XETA), based on the quantification of fluorescence-using the transgenic TH/bZip-eGFP *e.g.* [(*Tg(thibz:eGFP)*] line. Fifteen compounds were tested at different concentrations in presence of T_3_ 5 × 10^−9 ^M for 72 h. Scattered plots are shown with mean +/− SD of three to five independent experiments pooled (normalised on T_3_ to 100%). The GFP fluorescence in whole tadpoles (mainly heads) was measured and quantified after 72 h exposure. (**a**) Phenolic compounds: BPA, Triclosan and Benzophenone-3. (**b**) Phthalates: DBP and DEHP. (**c**) Organochlorine pesticides: HCB and 4′4-DDE. (**d**) Perfluorinated compounds: PFOA and PFOS. (**e**) Polyaromatic hydrocarbon: 2-Naphtol. (**f**) Halogenated compounds: Sodium perchlorate, PCB-153 and BDE-209. (**g**) Metals: Methylmercury and Lead chloride. Red arrowheads indicate concentrations of chemicals used in mix 1x (Table S1). Statistics were done with non-parametric Kruskal-Wallis test (*p < 0.05, **p < 0.01, ***p < 0.001, ****p < 0.0001). Hashes (###) represent *p* < 0.001, T_3_ vs Control using column to column comparison *(non parametric Mann Whitney).*

**Figure 2:**
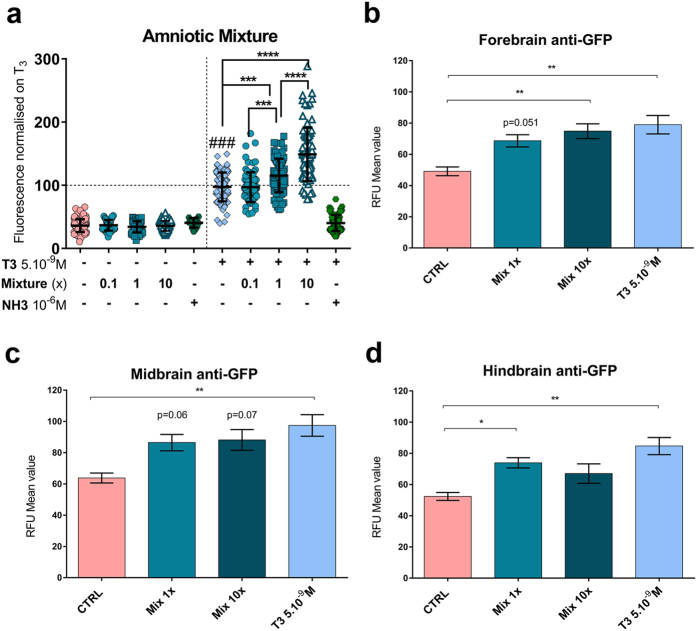
Thyroid disrupting activity of mixture assessed with XETA. Screening of thyroid disrupting activity of mixture of 15 molecules at concentrations measured in human amniotic fluid (mix 1x) 10 times more concentrated (mix 10x) and 10 times less concentrated (mix 0.1x) using *Tg(thibz:eGFP)* tadpoles. (**a**) GFP fluorescence (mainly localised in heads) of whole tadpoles exposed to mixture at 0.1x, 1x, 10x or a TR antagonist NH-3 (1 μM) with (right) or without (left) a T_3_ spike at 5 × 10^−9^ M. Quantification was done on images taken at 72 h exposure. Scattered dot plots are shown with mean +/− SD of five independent pooled experiments (normalised against T_3_). Statistics used non-parametric Kruskal-Wallis test (*p < 0.05, **p < 0.01, ***p < 0.001, ****p < 0.0001). Hashes (###) represent *p* < 0.001, T_3_ vs Control using column-to-column comparison. (**b–d**) Histograms represent mean (+/SEM) of relative fluorescence units (RFU) of GFP in forebrain (**b**), midbrain (**c**) and hindbrain (**d**) of tadpoles exposed to mixture for 72 h in the absence of T_3_. Regions were delimited manually on ventral brain images (see Fig. S2c). Statistics used non parametric Kruskal Wallis compared to CTRL.

In all experiments *X. laevis* tadpoles at stage NF45^16^ (non-feeding stage corresponding to one week post fertilisation development), were exposed for 72 h (at 23 °C), at which point they reached stage NF46/47. At this latter stage the thyroid gland starts to be functional. In humans, the thyroid gland becomes functional around 3 to 4 months of foetal life. Thus our exposure period corresponds to a period of human foetal development where only maternal thyroid hormone is available. In our model, the maternal thyroid hormone source is present in the yolk. Each chemical was screened in XETA at least at three concentrations, both alone (Fig. S1a–g) and against a tri-iodothyronine (T_3_) challenge (5 × 10^−9 ^M, Fig. 1). The T_3_ spike stimulates production of TRß (Thyroid Hormone Receptor Beta) that is inducible at this stage (ref. 17 and Fig. 2e), thereby amplifying responses (compare Fig. 1 and S1). The dose response relationships tested covered ranges found in human fluids, maternal blood or urine, cord blood serum or amniotic fluid (Table S1 and references therein). Note that Table S1 gives concentrations in molarity and μg/L as both units are commonly used in relevant studies.

Eleven of the 15 chemicals screened were positively identified as Thyroid Disruptors (TDs). Among the phenolic compounds tested, triclosan (TCS, an anti-microbial) significantly disrupted thyroid hormone signalling at 10^−7 ^M (Fig. 1a). Two phthalates (plastic softeners) were tested: dibutyl phthalate (DBP) and diethylhexyl phthalate (DEHP) (Fig. 1b). DEHP showed significant TD effects at 10^−7 ^M, in the range of human amniotic fluid levels. Both organochlorine pesticides tested, hexachlorobenzene (HCB) and 4-4′ dichlorodiphenyldichloroethylene (DDE, the main metabolite of DDT) (Fig. 1c), increased fluorescence from 10^−9 ^M and 10^−12 ^M onwards respectively. HCB significantly increased GFP at 10^−9 ^M, 10^−8 ^M and 10^−6 ^M. A non-monotonic, inverted ‘U’-shaped dose response was observed with the surfactant perfluorooctanesulfonic acid (PFOS) (Fig. 1d), with activation (*p* < 0.05) at 10^−10 ^M and inhibition (*p* < 0.001) at 10^−5 ^M. Perfluorooctanoic acid (PFOA) (Fig. 1d) enhanced transcriptional activity from 10^−10 ^M to 10^−6 ^M. All the other halogenated compounds had significant TD effects at the highest doses tested (Fig. 1f): perchlorate inhibited (10^−7 ^M) while a polychlorinated biphenyl (PCB-153) and a decabromodiphenyl ether (BDE-209) activated GFP at 10^−6 ^M. Two environmentally relevant heavy metals, known for their neurotoxic effects, were also screened (Fig. 1g). Methyl mercury significantly increased fluorescent at 10^−7 ^M, whereas lead chloride induced a significant decrease at 10^−7 ^M.

### A mixture of common chemicals dose-dependently disrupts thyroid hormone signalling during early brain development

Synergism of apparently inactive compounds has been reported^10^,^15^. We established a mixture (mix 1x) of each of these 15 chemicals at concentrations reported in human amniotic fluid (Fig. 1a–g (red arrowheads), Table S1) and tested it at 0.1x, 1x and 10x concentrations. Exposure of GFP-reporter tadpoles to the mixture induced a dose-dependent increase in fluorescence, by 18% (1x) and 49% (10x) compared to T_3_ alone suggesting increased T_3_ bioavailability (Fig. 2a). Exposure to mix 0.1x had no significant effect in XETA. The T_3_ dependency of the effects was confirmed using a T_3_ antagonist NH-3^18^ (Fig. S2a,b). NH-3 reduced the GFP signal induced by mix 10x both in the absence and the presence of T_3_ (Fig. S2a, left and right panel). In the case of mix 10x tested in the presence of the T_3_ spike, the GFP response was fully abrogated (Fig. S2a, right panel). Without the T_3_ spike, no significant modification of fluorescence was detected at the level of the whole tadpole for any concentration of the mixture nor for any single chemical, other than for BDE-209 at 10^−6 ^M (Fig. S1f). However, interference from epidermis and skull could mask brain specific responses. To determine whether mixture exposure affected brain GFP expression, we dissected the brains from tadpoles that had been exposed to mixture in the absence of exogenously added T_3_ and carried out anti-GFP immunohistochemistry. As indicated in Fig. 2b–d and S2c, a significant increase in fluorescence was measured. Notably, when signal intensity was analysed according to brain region (region of interest forebrain, midbrain or hindbrain) exposure to mix 1x and 10x increased GFP in the hindbrain and the forebrain respectively. The most marked effects were found in the forebrain, where GFP levels in mix 1x and 10x exposure were significantly increased (p = 0.05, and p < 0.01, respectively), showing brain region specific action of this chemical mixture.

### Exposure to chemical mixture modifies thyroid hormone-dependent and neuronal development-related gene expression in brain

**Figure 3:**
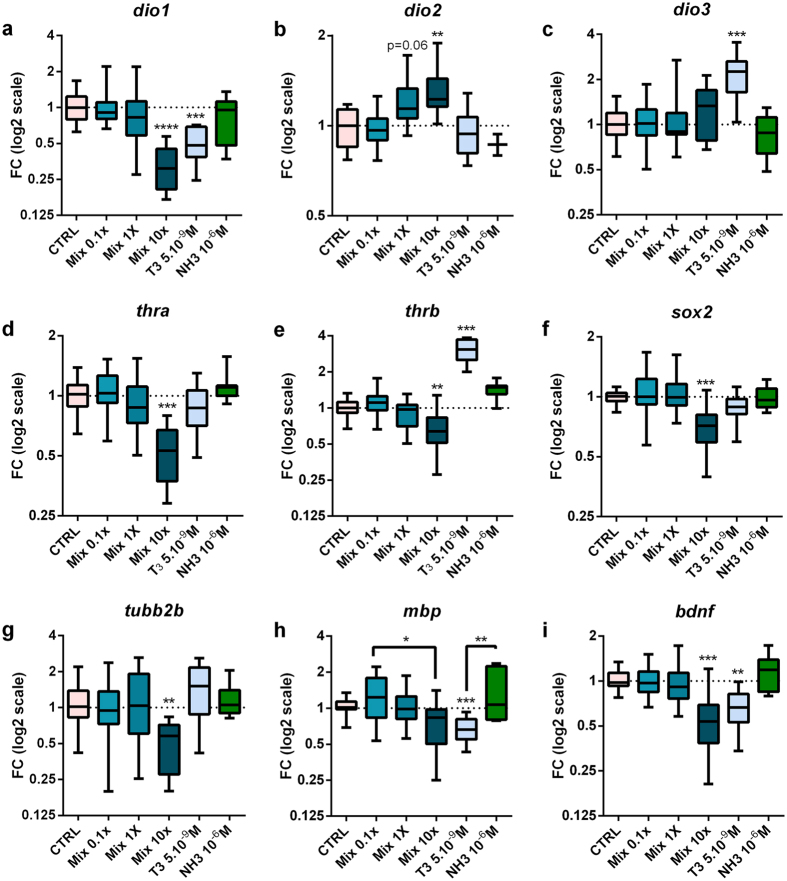
Mixture exposure modifies thyroid hormone and neuronal development related gene expression in brain. Wild type NF45 *X. laevis* tadpoles were exposed to mixture for 72 h in the absence of T_3_. For each concentration tested between 7 to 12 pools of three brains were used from at least four independent experiments. Total brain mRNA transcripts levels were quantified using RT-qPCR: (**a**), *dio1* (**b**), *dio2* (**c**), *dio3* (**d**), *thra* (**e**), *thrb* (**f**), *sox2* (**g**), *tubb2b* (**h**)*, mbp* (**i**)*, bdnf*. Relative fold changes were calculated using geometric mean of *ef1a* and *odc* as normalizers. Results are presented as fold changes using a log2 scale and DMSO-treated animals (CTRL) values for the 1.0 reference. Statistics were done on dCts and used Kruskal-Wallis tests (Box plots median and quartiles), *p < 0.05, **p < 0.01, ***p < 0.001, ****p < 0.0001.

**Figure 4:**
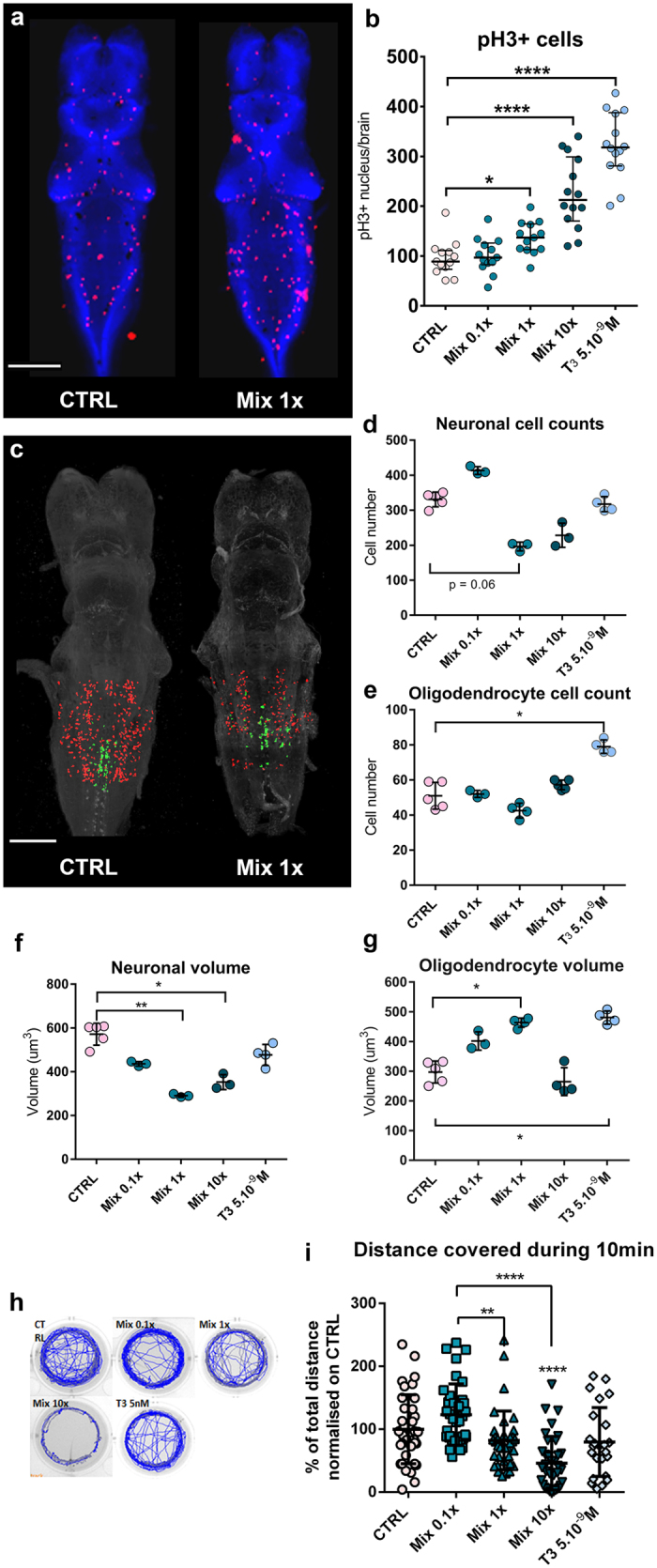
Mixture enhances proliferation in brain, modifies neural cell populations and behaviour. (**a**) Dorsal views of brains of mixture exposed wild type NF45 *X. laevis* tadpoles. Immunohistochemistry used the anti-PH3 antibody (red, mitosis) and DAPI (blue, nucleus). Scale bar, 200 μm. (**b**) Numbers of proliferating cells in (PH3 + cells) in tadpole brains following mixture exposure. n = 13 brains per condition, 5 independent experiments pooled (representative number of positive cells in each brain). Statistics used 2-way ANOVA and Dunnett’s post-test (Medians ± SDs, *p < 0.05,****p < 0.0001) (**c**) CLARITY imaging illustrating the region of interest delimited for analysis in (**d**–**g**) on hind brain. Examples of control (left) and mix 1x exposed (right) double transgenic tadpoles *Tg(nβt:DSRED)* (neurons, red) and *Tg(Mmu.mbp:NTR-eGFP)* (oligodendrocyte, green). Scale bar, 200 μm. (**d–g**) Quantification of CLARITY signals obtained for each fluorescent signal in hindbrain. Neuron (**d**) and oligodendrocyte (**e**) numbers and cell volumes (**f**,**g**), n = 3 to 5 brains, Statistics used non parametric Kruskal Wallis ANOVA and Dunn’s post-test (Means ± SDs, *p < 0.05, **p < 0.01) (**h**) Wild type NF45 *X. laevis* tadpoles were exposed to DMSO (CTRL), or mixture (0.1x, 1x, 10x), T_3_ 5 × 10^−9^ M for 72 h for mobility analysis. Example of total distance covered in 10 mins under 30 secs/30 secs light (blue lines)/dark (grey lines) cycles by one tadpole per condition (**i**) Mean distance covered during 10 mins under different conditions. Distance is normalized versus controls for 4 independent experiments with n = 12 per experiment. Representation uses scattered dot plots Mean +/− SD. Statistics used meta-analysis with Kruskal-Wallis. Note that stars directly over a group indicates significant difference with CTRL group (Error bars indicate s.e.m, **p < 0.01, ****p < 0.0001).

Having established that TD effects were measurable in brains without an exogenous T_3_ spike, we next examined effects of exposure on brain development using the mixture without T_3_ co-treatment, thereby relying on endogenous T_3_ signalling (Figs 3 and 4, S3–S6). qPCR was used to examine gene expression in dissected brains (Fig. 3, S3, S4) following exposure to the mixture, T_3_ (5.10^−9 ^M) or NH-3 (10^−6 ^M). Mixture exposure (72 h) modified expression of multiple genes, including those encoding the deiodinases (enzymes that determine T_3_ bioavailability)^19^, thyroid hormone receptors (TRs), thyroid hormone transporters (THTs) and genes implicated in neural stem cell renewal and neuronal differentiation. Expression of *dio1,* encoding deiodinase1 (D1, an activating or inactivating deiodinase)^20^, was significantly decreased, whilst expression of *dio2,* encoding deiodinase2 (D2, an activating enzyme) was increased (Fig. 3a,b) as suggested in Fig. S1h (T_4_ co-treatment). Genes encoding THTs were also significantly modulated by exposure to mixture (Fig. S4m–q). Expression of both TRα and TRβ mRNA was significantly down-regulated by exposure to mix 10x (Fig. 3d,e). Amongst the T_3_-target genes affected by exposure to mix 10x, when compared to mix 0.1x, were the pluripotency gene *sox2* (Fig. 3f), the neurotrophic factor *bdnf* (Fig. 3i) and genes implicated in neuronal and oligodendrocyte differentiation, namely *tubulin2b, mecp2, dcx* (Fig. 3g and S3b) and *mbp* (Fig. 3h). Addition of exogenous T_3_ resulted in similar or amplified expression of T_3_ target genes (Fig. S3).

### Chemical mixture enhances cell proliferation in brain and modifies neural cell populations

Modulation of neural differentiation genes suggests that exposure to the chemical mixture could affect progenitor proliferation as well as neuronal and glial cell numbers. We verified whether this was the case using immunocytochemistry for phosphorylated histone H3 (P-H3), a mitotic marker, on mixture-exposed brains^21^. Exposure induced a dose-dependent increase in proliferating cells (PH3+ cells), from 96 ± 10 (mean ± SEM) to 136 ± 9 with mix 1x and 225 ± 19 with mix 10x (Fig. 4a,b).

To further examine if neural lineage decisions were modified by mixture exposure, dissected brains were subjected to CLARITY^22^ in order to visualize the whole brain (Videos S5a,b,c) and quantify neuronal and oligodendrocyte populations, as well as individual cell volumes (Fig. 4c–g). We used double transgenic tadpoles *Tg(Mmu.mbp*:GFP,*nβt:*DsRed), in which fluorescent markers label mature oligodendrocytes in green and differentiated neurons in red. The oligodendrocyte marker (GFP) is driven by myelin basic protein (*mbp*) regulatory elements from mouse^23^. The neuronal marker (DsREd) is driven by neural β tubulin (*nβt*) regulating elements^24^. See material and methods and Fig. 4c for further explanations. Disruption to thyroid hormone signalling was measured in the hindbrain (Fig. 2f), where both oligodendrocytes and neurons are present. Here exposure to mix 1x induced a decrease in differentiated neuron numbers that almost reached significance (Fig. 4d, *p* = 0.06). Neuron and oligodendrocyte volumes were inversely affected with exposure to mix 1x, decreasing neuron volume significantly while increasing that of oligodendrocytes (*p* < 0.01 and *p* < 0.05 respectively, Fig. 4f,g).

### Chemical mixture exposure dose-dependently alters tadpole mobility

We next addressed the phenotypic consequences of short-term (72 h) exposure since embryonic motor behaviour changes can indicate small modifications in the neural circuitry controlling movement^25^. For this purpose, we used a video tracking system and recorded the total distance covered by individual tadpoles with 30 secs alternating light and dark cycles for total of 10 mins (Fig. 4h–g, S6a–c and Videos S6,d,f). Figure 4h shows representative traces of single tadpoles exposed to different concentrations of mixture or T_3_ (5.10^−9 ^M). The total distance travelled decreased dose-dependently with increasing mixture concentration and in the case of mix 10x, by more than 50% (*p* < 0.0001) (Fig. 4i). These differences were observed over the 10 minutes tracking (Fig. S6b) and independently of light or dark periods even though tadpoles are stimulated by light (Fig. S6c).

## Discussion

There has been a 300-fold increase in the number and quantity of chemicals released into the environment in the last 50 years^26^. Many chemicals are now ubiquitous in the environment and in humans, including in pregnant women^6^. In this study, the rationale was to select chemicals found ubiquitously, test their thyroid disrupting effects individually and then, to represent intra-uterine exposure, test a mixture at concentrations found in amniotic fluid.

### Focus on Thyroid disruption

We emphasised disruption of thyroid hormone signalling because (i) of the tight dependence of brain development on maternal levels of thyroid hormone^4^ (ii) a 3 day screening assay undergoing OECD validation is available and (iii) this hormonal axis is highly sensitive to endocrine disruption^27^. This sensitivity could relate to the complexity of thyroid hormone production that includes iodine uptake through specific symporters and a highly-regulated organification process^28^, but also to specific enzymes controlling thyroid hormone availability in peripheral targets^1^. A salient point is that thyroid hormones are the most complex halogenated molecules produced by vertebrates and the only one to contain iodine. Interestingly, eight of the 15 compounds tested here are halogenated, all of which showed thyroid signalling disruption, while most non-halogenated molecules (*i.e.* BPA, napthol and benzophenone-3) were inactive in the XETA test at relevant concentrations. Only two non-halogenated molecules were active: mercury and lead. Mercury chelates selenium^29^, an element required for synthesis and activity of all deiodinases^30^. This feature links mercury to interference with thyroid hormone activation/inactivation via deiodination, through selenocysteine deiodinases that are active in Xenopus tadpoles at this developmental stage^20^,^31^. These enzymes finely tune the bioavailability of the active form of thyroid hormone (T_3_) in each cell.

### Increased TH signalling with individual compounds and mixtures

These deiodination processes, and more generally the complexity of thyroid hormone signalling, could also contribute to explaining why the majority of individual chemicals and the mixture induced increase in thyroid hormone availability (after 72 h). It is worth pointing out that we did not observe additive effects of the individual compounds used as a mixture. Indeed, at the concentrations used in the mixture, certain compounds could be exerting negative effects whilst other exert positive effects. Given the multiple possible pathways affected, the overall readout on transcription could be muted. Similarly, the effect of one compound could override that of others. Previous epidemiological and experimental data on some individual chemicals could have led to predict decreased thyroid hormone availability. For instance, high PCB or BDE exposure depresses circulating thyroid hormone levels in humans and different species^32^,^33^. However, many of these experiments were based on long term exposure where increased clearance is subsequent to transient interactions with distributor proteins. In such cases, one could well expect a transient increase in thyroid hormone availability as observed in our experimental model^34^. Moreover, brain T_3_ increased bioavailability is also strongly suggested by the mixture-induced increased expression of *dio2* encoding for activating enzyme D2 (Fig. 2b). In this light, it should be borne in mind that our *in vivo* screening readout encompasses multiple levels of thyroid hormone signalling axis which could, by definition, indicate multiple levels of disruption. Indeed, this screening model offers the huge advantage of detecting chemicals interacting directly or indirectly with TH signalling whatever the level of disruption, but requires deeper investigations to identify specific mode of action. The thyroid disruption property of the mixture and the subsequent fluorescence increase, while possibly indirect for certain components, is clearly shown by the co-exposure with antagonist NH-3 that abrogated 10x mixture induced fluorescence in presence or absence of T_3_ (Fig. S2a and b).

Early stages of development are considered to be amongst the most vulnerable windows for exposure as they represent ongoing organogenesis^35^. Studying mammalian embryos at these early stages is challenging due to their intrauterine development and limited numbers of embryos per mother. Hence, there is a need for more tractable models. The free-living amphibian *X. laevis* tadpole, provides large-scale screening tools and allows easy access to early developmental stages. A further advantage of the Xenopus system is the high homology of thyroid hormone signalling with mammals that is not fully shared by other free-living aquatic models such as teleosts.

After having established the thyroid disrupting effect of the mixture, rationale was then to mimic an embryonic exposure during a critical period for brain development. For that purpose mixture alone was applied to embryos before thyroid gland formation during neurogenesis. Our results show that mixture exposure affects T_3_–dependent transcription, cellular responses and behaviour. These multiple early developmental effects could well be interrelated.

Among the brain-expressed genes significantly modified were numerous actors implicated in thyroid hormone signalling, such as deiodinases, TRs, thyroid hormone transporters, and thyroid hormone targets including determinants of neural development. Expression of the activating/inactivating deiodinase, D1 was significantly decreased, whilst that of the activating deiodinase, D2, was significantly increased. These findings fit with the results from the XETA test that displayed a dose-dependent increase in thyroid hormone signalling following chemical mixture exposure, reflecting greater bioavailability of the hormone.

### Relevance of these results to human brain development

At first sight, given the essential role of thyroid hormones in brain development, one might think that more hormone is not problematic. However, a number of results counter this idea. First, Korevaar *et al*.^4^ in their study of mother/child pairs, showed that maternal hyperthyroidism has an equally adverse effect on children’s IQ and brain structure, as does maternal hypothyroidism. Second, many rodent studies have revealed deleterious effects of hyperthyroidism and hypothyroidism during brain development^36^. Finally, during neurogenesis, thyroid hormones act as differentiation signals, directly repressing the pluripotency gene *Sox2*^37^. Early exposure to excess thyroid hormone could therefore induce precocious differentiation of the neural progenitor populations, with ensuing modifications of brain size and organisation. In the present study, expression of *sox2* was down-regulated by exposure to mix 10x, as was the expression of a number of neural markers, including markers of neuronal (*tubb2b*) and oligodendrocyte (*mbp*) differentiation. Similarly, expression of an essential nerve growth factor, *bdnf*, was significantly decreased. BDNF variants have repeatedly been linked in human studies to autism spectrum disorder (ASD)^38^,^39^ as well as animal models of this neurodevelopmental disorder^40^,^41^. In our model, most of the changes in brain gene expression were only seen following exposure to mix 10x. However, it should be borne in mind that in this experimental context exposure is limited to 72 h. During this time it is probable that many of the chemicals are catabolised by the tadpoles, which are metabolically competent at this stage^42^. A similar situation is expected in the uterine environment, but in this case metabolites can accumulate in the amniotic fluid and prolong exposure if not removed through the umbilical cord blood and excreted by the mother. Moreover, from a legislative point of view, tolerable daily intake is calculated from no observed adverse effect level (NOAEL) in animal models with a security factor of 100 (10 for intra and 10 for interspecies differences). This also means that any result obtained in an animal model with 10 times human levels is relevant and highlight the absolute need for a better legislation.

Strikingly, in tadpoles exposed to mixture, we found that exposure to the chemical mixture at 1x concentration significantly increased proliferation in neurogenic zones (Fig. 4a,b) but also oligodendrocyte volume (Fig. 4g) whilst decreasing that of neurons (Fig. 4f). Interestingly, autopsies of brains from ASD patients have revealed changes in neuronal cell volumes^43^,^44^. Changes were also found in ratios of oligodendrocyte to neurons, with numbers of neurons being significantly reduced following short-term exposure to the chemical mixture (Fig. 4d). Again, this finding has relevance to human data. Analyses of maternal thyroid hormone levels during early pregnancy revealed that both maternal hypothyroidism and hyperthyroidism can result in changes in children’s brain structure with modifications of grey to white matter ratios^4^, reflecting changes in neuron to oligodendrocyte numbers.

Finally, we found that the molecular and cellular modifications resulting from mixture exposure led to marked behavioural changes as assessed by mobility tracking. Exposure to mixture significantly reduced total distance travelled by tadpoles, with mix 10x reducing distance travelled by over 50%. Maternal hypothyroidism increases the risk for many neurodevelopmental diseases characterised by behavioural problems, including ASD and Attention Deficit Hyperactivity Disorder (ADHD)^45^,^46^. We provide evidence that most of the ubiquitous compounds measured in human amniotic fluid disrupt thyroid signalling alone or if applied as a mixture. We also show that mixture exposure results in a number of T_3_ –like effects in expression of key genes and neural proliferation in the brain with ensuing effects on behaviour.

Many chemicals in this mixture could also affect other endocrine pathways beside thyroid hormone. An example is phthalates that are known to affect androgen signalling^47^ but exposure to which has also recently been linked during pregnancy with altered maternal thyroid levels^48^. Importantly, epidemiological studies show that maternal exposure to many of the chemicals studied here can affect offspring IQ and/or neurodevelopmental disease risk. This is the case for PCBs that have been linked to IQ loss^49^ and increased ADHD risk^50^. As to phthalates, numerous members of this vast chemical category, have also been linked to IQ loss^51^ and risk for different forms of neurodevelopmental disease^52^,^53^. Further, multiple studies show that increased maternal perchlorate levels correlate negatively with offspring IQ^54^. Lastly, PBDEs represent yet another large chemical category where maternal exposure has repeatedly been associated with IQ loss and increased ASD risk^55^.

## Conclusions

The above results demonstrate that early embryonic exposure to a mixture of common chemicals alters thyroid hormone signalling, brain structure and behaviour. These findings can be placed in the context of recent epidemiological studies showing that small variations in maternal thyroid hormone during early pregnancy impact children’s IQ^4^. Our results would thus argue for an urgent revisiting of the regulatory scenario used to determine how common chemicals and their mixtures affect human health.

## Material and Methods

### *Xenopus laevis* strains and rearing

*Xenopus laevis* strains were maintained in accordance with institutional and European guidelines (2010/63/UE Directive 2010), all procedures and methods used are following institutional and European guidelines (2010/63/UE Directive 2010) and have been approved by the local ethic committee (Cometh) under the project authorization No. 68-039. The transgenic X*. laevis* lines used were; *Tg(thibz:eGFP)* (homozygous)^14^ and a double transgenic (heterozygous) *Tg(Mmu.mbp:NTR-eGFP,nβt:DSRED)* obtained by crossing *Tg(Mmu.mbp:NTR-eGFP)*^23^ with *Tg(nβt:DSRED)*^24^. In *Tg(thibz:eGFP)* GFP is expressed under the control of 850 bp of the regulatory region of THbZIP transcription factor, a TH regulated gene. In *Tg(Mmu.mbp:NTR-eGFP),* an oligodendrocyte marker (GFP) and an enzyme nitroreductase (NTR) are driven by myelin basic protein (*mbp*) regulatory elements from mouse. Note that nitroreductase can specifically induce apoptosis in oligodendrocytes^23^, but we did not use this property in this study. In *Tg(nβt:DSRED)*, the neuronal marker (DsRED) is driven by neuronal β tubulin (*nβt*) regulating elements. Tadpoles were obtained by natural breeding between wild type (WT) and/or transgenic animals and raised as described^12^.

### Chemicals

The following chemicals were purchased from Sigma-Aldrich (Saint-Quentin Fallavier, France): bisphenol-A (BPA purity >99%), triclosan (TCS >97%), benzophenone-3 (BP3_98%), decabromodiphenyl ether (BDE-209 98%), sodium perchlorate (>98%), 4-4′-dichlorodiphenyldichloroethylen (4,4′-DDE 99%), hexachlorobenzene (HCB 99.9%), dibutylphtalate (DBP 99%), diethylhexylphtalate (DEHP 99%), PCB-153, 2-Naphtol (99%), perfluorooctanesulfonic acid (PFOS >98%), perfluorooctanoic acid (PFOA >98%), methyl mercury chloride (>99.9%), lead chloride (98%), dimethyl sulfoxide (DMSO), acetone, 3,3′,5,5′tetraiodo-L-thyronine T_4_ >98%) and triiodothyronine (T_3_ 99%). NH-3, a thyroid hormone antagonist^18^ was synthesized by AGV Discovery (France), absence of contamination by benzofurane was verified^56^. All chemicals were dissolved at 10^−1^ M in DMSO, with the exception of HCB (10^−1^ M in acetone) and BDE 209 (10^−2 ^M in DMSO). These solutions were aliquoted and stored at −20 °C until use. T_3_ was prepared in 30% NaOH, 70% milliQ Water at 10^−2 ^M concentration, aliquoted and stored at −20 °C until use. The chemical mixture was prepared at 10^5^-fold concentration by mixing appropriate volumes of stocks as described in Supplementary Table 1, aliquoted and stored at −20 °C.

### Chemical exposure protocol for Xenopus embryonic thyroid assay (XETA) screening

Screening for thyroid disrupting chemicals was carried out using XETA as previously described using stage NF45 tadpoles, from *Tg(thibz:eGFP)* transgenic *X. laevis* at stage NF45 (1 week old)^12^. Fifteen tadpoles were placed per well in 6 well plates (TPP Switzerland), containing either control solvent (DMSO) or chemical. DMSO concentration was 0.01% in all treatments and the pH remained unchanged (Fig. S2). Plates were placed at 23 °C for 3 days. The chemical solutions were renewed every day, at regular 24 h intervals. After 72 h exposure, tadpoles were anesthetized with 0.01% MS-222 and placed dorsally, one per well in a black, conic based-96 well plate (Greiner). Images were acquired with a 25x objective and 3 s exposure using an Olympus AX-70 binocular equipped with long pass GFP filters and a Q-Imaging Exi Aqa video camera. QC Capture pro (QImaging) software was used for image acquisitions and quantifications were carried out using ImageJ. All pictures of a group (chemical and concentration) were stacked, the 3 layers of RGB pictures were split and the red and blue channels subtracted from the green channel to exclude non-specific signals (integrated density of all images were used). Quantifications were carried out on whole pictures and data was expressed in relative units of fluorescence (RFU). All values were normalized to the T_3_ group (100%). GraphPad Prism 6 software was used for graphs and statistical analysis.

### Statistical analysis for XETA

Results are presented as scatter dot blots with mean +/− SD. Experiments were validated with a column to column comparison between the control and T_3_ group using the non-parametric Mann-Whitney test. T_3_ spiked and non-spiked mode were analysed separately using non-parametric Kruskal-Wallis’ followed by Dunn’s test. Differences were considered significant at p < 0.05(*), p < 0.01 (**), p < 0.001(***) and p < 0.0001(****).

### RNA extraction and gene expression analysis

For gene expression analysis, wild type *X. laevis* tadpoles were subjected for 72 h to chemicals as previously described above. After 72 h, tadpoles were anesthetized in 0.01% MS-222, and brains dissected on ice under sterile conditions. Two different RNA extraction methods were used and gave comparable results. Initially brains were dissected and a pool of three brains were placed in 1.5 ml Sorenson tubes, 4 tubes per condition (control, chemically treated), flash frozen in liquid nitrogen and stored at −80 °C. RNA extraction used QIAGEN RNeasy micro plus kits following the manufacturer’s recommendations. The second method used a pool of two dissected brains placed in 1.5 ml Sorenson tubes containing 100 μl lysis buffer (provided in RNAqueous micro kit (Ambion)), 5 tubes per condition (control, chemically treated etc.) flash frozen in liquid nitrogen and stored at −80 °C. RNA extraction used RNAqueous Micro kit (AMBION, ThermoFischer). RNA concentrations were determined using a spectrophotometer (NanoDrop ThermoScientific, Rockford, IL) and RNA quality verified using BioAnalyzer (Agilent) where we only validated samples with RIN >8. Total extracted RNA (500 ng) was used for reverse transcription using a High Capacity cDNA RT kit (Applied BioSystem, Foster City, CA). The single stranded complementary DNA (cDNA) obtained was used as a template for qPCR.

Quantitative PCR was carried out using QuantStudio 6 flex (Life technologies) on 384 well-plates, with a standard reaction per well containing 1/20 diluted cDNA as template (1 μl per well) plus 5 μl of mix (Power SyBR mix, Applied BioSystem). Relative concentrations of cDNA were calculated by the 2^−ΔΔCt^ method^57^ for the analysis of relative changes in gene expression. For normalizing, a geometric mean of endogenous controls (elongation factor alpha (*ef1alpha*) and ornithine decarboxylase (*odc*)), were used as the two reference genes.

### Statistical analysis for qPCR

Data are presented as fold change (2^−ΔΔCt^) using a log (base2) scale plotted as a traditional box and whisker plot by Tukey where the bottom and top of the box represent the 25^th^ lower and 75^th^ percentile, and the median is the horizontal bar in the box. Statistical analyses were performed on delta Cts using non-parametric Kruskal Wallis’ test followed by Dunn’s post-test (all compared to the control group). Certain column to column comparisons were done when necessary and have been annotated in the figures accordingly. Significance was determined at p < 0.05(*), p < 0.01 (**) and p < 0.001(***).

### Immunohistochemistry (IHC) for cell proliferation

After 72 h exposure, tadpoles were euthanized in MS-222 1 g/l, fixed in 4% paraformaldehyde for 3 h at RT (room temperature), placed in PBS for either immediate use or in cryoprotectant and stored at −20 °C. Primary antibodies: anti-Ser10 phosphorylated on Histone H3, rabbit, (06–570 Millipore) or mouse (05–806 Millipore) used at 1/300 dilution for *in toto* immunohistochemistry. All positive nuclei were counted from 5 independent experiments with n = 2 to n = 5 per experiment and statistical analyses performed using two-way ANOVA followed by Dunnett’s post-test (all compared to control group). Significance was determined at p < 0.05(*) and p < 0.0001(****).

### Behaviour analysis –total distance covered

Mobility of NF45 tadpoles, exposed (72 h) to mixture (0.1x, 1x, 10x), T_3_ 5.10^−9^ M or solvent (DMSO) was assessed using the DanioVision (Noldus) behaviour analysis system. After an initial rinse, tadpoles of each group were placed one per well of a polypropylene transparent 12 well plate (TPP, Switzerland) in 4 ml of Evian water. Tadpoles were left to accommodate for 15 minutes before placing the plate in the Danio Vision Module. This module consists of an opaque box in which the plate holder under an infrared camera. Plates were recorded for 10 mins as a movie (example in videos S6 e–g). Light was used to stimulate movement *ie* periods of 30 seconds alternating light and dark cycles. Maximal light stimulus (5 K Lux) was used during light on. Distance travelled during the 10 mins was analysed using EthoVision software (11.5, Noldus, Wageningen, The Netherlands).

### Statistical analysis of mobility

Differences between control, mix 0.1x, mix 1x, mix 10x were analysed using the non-parametric Kruskal Wallis’ test. Differences were significant at *p* < 0.01 (**) and p < 0.0001(****).

### Clarification and immunohistochemistry (IHC) for neuronal and oligodendrocyte cell populations

Transgenic tadpoles were obtained from crossing the double transgenic *X. laevis* line carrying p*mbp*:NTR-eGFP and p*nβt*:DsRed with wildtype *X. laevis.* Fluorescent tadpoles were sorted at stage NF45 and exposed to chemical treatment as described previously. After 72 h treatment, the tadpoles were anaesthetized in MS222 1 g/l, fixed in 4% paraformaldehyde for 3 h at RT and stored (0.4% PFA, 4 °C) until clarification.

### CLARITY

Fixed tadpoles and dissected brains were subjected to clarification following the CLARITY protocol^58^ with some tissue-specific adaptations: samples were infused in a pre-cooled solution of freshly prepared hydrogel monomers (0.01 PBS, 0.25% VA-044 initiator (wt/vol), 5% dimethyl sulfoxide (vol/vol), 1% PFA (wt/vol), 4% acrylamide (wt/vol) and 0.0025% bis-acrylamide (wt/vol)) for 2d at 4 °C. After degassing the samples, hydrogel polymerization was triggered by replacing atmospheric oxygen with nitrogen in a desiccation chamber for 3 h at 37 °C. The superfluous hydrogel was rinsed off and samples transferred into embedding cassettes for lipid clearing. Passive lipid clearing was performed at 40 °C for 8 d in the clearing solution (8% SDS (wt/vol), 0.2 M boric acid, pH adjusted to 8.5) under gentle agitation. Subsequently, the samples were thoroughly washed in 0.01 M PBS, tween 0.1% (wt/vol, PBSt, RT, 2d) with gentle agitation.

### Immunostaining of clarified samples

CLARITY-processed brains were incubated in blocking solution (0.01 M PBS, 0.1% tween 20 (vol/vol), 1% TritonX100 (vol/vol), 10% dimethyl sulfoxide (vol/vol), 10% normal goat serum (vol/vol), 0.05 M glycine) overnight at 4 °C. Samples were further incubated in staining solution (0.01 M PBS, 0.1% tween 20 (vol/vol), 0.1% Triton X100 (vol/vol), 10% dimethyl sulfoxide (vol/vol), 2% normal goat serum (vol/vol), 0.05% azide (vol/vol)) with primary antibodies (chicken anti-GFP, Avès Labs, 1:400 and rabbit anti-DsRed, Clontech, 1:400) for 7d at RT under gentle agitation. After 2 washes with PBSt, samples were incubated in a staining solution with secondary antibody (goat anti-chicken Alexa Fluor 488, Invitrogen, 1:600 and goat anti-rabbit 555, Invitrogen, 1:600) for 7d at RT. Samples were then washed for 48 h in PBSt.

### Imaging using a high refractive index solution

A fructose-based high refractive index solution (fHRI) was prepared as follows; 70% fructose (wt/vol), 20% DMSO (wt/vol) in 0.002 M PBS, 0.005% sodium azide (wt/vol). The refractive index of the solution was adjusted to 1.4571 using a refractometer (Kruss). The clarified samples were incubated in 50% (vol/vol) fHRI for 6 h and further incubated in fHRI for >12 h. For imaging, samples were mounted in 1% (wt/vol) low melting point agarose and covered with fHRI. Whole-mount brain fluorescence was captured using a Leica TCS SP8 laser scanning confocal microscope equipped with a Leica HC FLUOTAR L 25x/1.00 IMM motCorr objective.

### Image treatment and statistical analysis

Image stacks were converted from their native 12 bit lif-format to series of 8bit-pngs using CLAHE (contrast limited adaptive histogram equalization, Zuiderfeld, 1994) for ImageJ (Rasband *et al*., http://rsbweb.nih.gov/ij/) as implemented in fiji (Saalfeld, http://fiji.sc/Enhance_Local_Contrast_%28CLAHE%29). The parameters for CLAHE were empirically tested and set to a block size of 127, 256 bins and a slope of 3 (default values). CLAHE enhances the contrast and intensity of weak signals significantly while not over-saturating strong signals. Images were analysed using Imaris software (Imaris software package, Bitplane AG, Zurich, Switzerland). The areas of individual neurons and oligodendrocytes were determined and used to calculate the mean size of individual cell volume. At least 3 brains from independent experiments were used for each experiment shown.

## Additional Information

**How to cite this article**: Fini, J.-B. *et al*. Human amniotic fluid contaminants alter thyroid hormone signalling and early brain development in Xenopus embryos. *Sci. Rep.* **7**, 43786; doi: 10.1038/srep43786 (2017).

**Publisher's note:** Springer Nature remains neutral with regard to jurisdictional claims in published maps and institutional affiliations.

## Supplementary Material

Supplementary Information

Supplementary Figure S5a

Supplementary Figure S5b

Supplementary Figure S5c

Supplementary Figure S6d

Supplementary Figure S6e

Supplementary Figure S6f
