# Supplementary material for: Human amniotic fluid contaminants alter thyroid hormone signalling and early brain development in Xenopus embryos
Source: Sci Rep. 2017 Mar 7;7:43786. doi: 10.1038/srep43786 (PMC5339866; doi:10.1038/srep43786)
Supplement: Supplementary Information [file srep43786-s1.pdf]

Supplementary information:

Human amniotic fluid contaminants alter thyroid hormone signalling and early brain development in Xenopus embryos

Jean-Baptiste Fini*[1], Bilal B. Mughal*[1], Sébastien Le Mével[1], Michelle Leemans[1], Mélodie Lettmann[1], Petra Špirhanzlová[1], Pierre Affaticati[2], Arnim Jenett[2] and Barbara A. Demeneix[†1]

Supplementary Figure legends

Supplementary Figures

Primer list

Supplementary Table1 + Supplementary References

Supplementary Figures:

- **Supporting information for Figure 1**
  - Figure. S1 - (Figure 1 extended data) Thyroid disrupting activity of individual chemicals and mixture assessed with XETA without T_3 as a spike and mixture with a T_4 spike
- **Supporting information for Figure 2**
  - Figure S2a - NH3 abrogates GFP fluorescence induced by T_3 + Mixture 10x
  - Figure S2b - NH3 significantly reduces GFP fluorescence in Mix 10x exposed tadpoles.
  - Figure S2c - Anti-GFP using immunohistochemistry on *Tg(thibz:eGFP)* tadpoles brains is increased by mixture exposure in absence of T_3
  - Figure S2d- No change in pH observed with either T_3 or mixture

- **Supporting information for Figure 3**
  - Figure S3 (Figure 3 extended data) Mixture exposure modifies thyroid hormone and neuronal development related gene expression in brain when using T_3 as a spike
  - Figure S4 (Figure 3 extended data) Mixture exposure modifies neuronal development related gene expression in brain in the absence of a T_3 spike.

- **Supporting information for Figure 4**
  - Figure S5 - 3D brain video (CLARITY)
    a) Wild type whole brain
    b) T_3 treated whole brain
    c) Mix 10x treated whole brain
  - Figure S6 (BEHAVIOUR)
    a) Protocol (schematic) for assessing locomotor activity
    b) Distance covered by tadpoles over 10 minutes (detail minute by minute)
    c) Distances covered by tadpoles with regards to dark and light periods.
    d) Mobility video (Control): Full length video of the 12 well plate subjected to mobility assay
    e) Mobility video (T_3): Full length video of the 12 well plate subjected to mobility assay
    f) Mobility video (Mixture 10x): Full length video of the 12 well plate subjected to mobility assay

Supplementary figure legends

Figure. S1: **Thyroid disrupting activity of individual chemicals and mixture assessed with XETA without T_3 as a spike and mixture with a T_4 spike (*Figure 1 extended data*):** Screening of thyroid disrupting activity of molecules measured in humans with the Xenopus Embryonic Thyroid Assay (XETA), based on the quantification of fluorescence-using transgenic Thbzip-eGFP animals. Fifteen compounds were tested at different concentrations. Scattered plots are shown with mean+/- SD of three to five independent experiments pooled. The GFP fluorescence was measured and quantified after 72h exposureChemicals tested alone without a T_3 spike. **a**, Phenolic compounds: BPA, Triclosan and Benzophenone-3. **b**, Phthalates: DBP and DEHP. **c,** Organochlorine pesticides: HCB and 4'4-DDE. **d,** Perfluorinated compounds: PFOA and PFOS. **e,** Polyaromatic hydrocarbons: 2-Naphtol. **f,** Halogenated compounds: Sodium perchlorate, PCB-153 and BDE-209. **g,** Metals: Methylmercury and Lead chloride. **h,** Exposure to mixed compounds alone and against T_4 10nM spike (**a-g**). Results represent 3 pooled experiments, normalised against T_3 or T_4 values. Statistics used meta-analysis with Kruskal-Wallis (Mean ± SDs, *$p < 0.05$, ****$p < 0.0001$). Hashes (###) represent $p < 0.001$, T_4 vs Control.

Figure. S2 ***(Figure 2 extended data)***

a) **NH3 abrogates GFP fluorescence induced by T3 + Mixture 10x**. *Tg(thibz:eGFP)* tadpoles were exposed for 3d to Mix 1x or 10x (see table S1 for exact composition), NH3 2µM with or without T_3 5nM for 72h. Quantification of fluorescence was done on whole tadpoles' head as illustrated.

b) NH3 significantly reduces GFP fluorescence in Mix 10x exposed tadpoles. Results of Fig S2a in absence of T_3 spike, analyzed with a specific quantification of tadpole brain fluorescence as illustrated.

c) Anti-GFP using immunohistochemistry on Tg(thibz:eGFP) tadpoles brains is increased by mixture exposure in absence of T3. *Tg(thibz:eGFP)* tadpoles were exposed for three days to either Mix 1x, 10x or T_3 5nM. Brains were dissected after 3h fixation in PFA 4% at RT and stored in cryoprotectant until being subjected to IHC with the chicken anti eGFP antibody. Left panel represents representative brains in ventral view for each group. Regions of interest (ROI) in brain: Forebrain (FB), Midbrain (MB, Hindbrain (HB) are outlined with hashed lines. Scale bar 200µm. Right panel represents GFP quantification on the whole brain.

d) No observable change in water pH following addition of either T_3 or mixture. pH stripes (Fisher brand) with either control water (Evian + 1/10000 DMSO), T_3 5.10^{-9}M (same concentration of DMSO as in control) or mixture at 0.1x, 1x or 10x concentration.

Figure. S3: (*Figure 3 extended data)* Mixture exposure modifies thyroid hormone and neuronal development related gene expression in brain when using T_3 as a spike. Mixture exposure modifies thyroid hormone and neuronal development related gene expression in brain when using T_3 as a spike: RT-qPCR for **a**, *dio1* **b**, *dio2* **c**, *dio3* **d**, *thra* **e**, *thrb* **f**, *sox2* **g**, *tubb2b* **h**, *mbp* and **i**, *bdnf* **j** *thibz* **k**, *klf9,* **l** *mecp2,* **m** *oatp1c1,* **n** *mct8,* **o** *mct10,* **p** *lat1* and **q** *lat2.*

Relative fold change presented using log(2)scale against T_3. Statistics used one way non parametric Kruskal-Wallis test (Medians, Whiskers boxes mean and max, *$p < 0.05$, **$p < 0.01$, ***$p < 0.001$, ****$p < 0.0001$).

Figure S4 ***(Figure 3 extended data)*** **Mixture exposure modifies additional thyroid hormone and neuronal development related gene expression in brain: RT-qPCR** for **a,** *klf9* **b**, *cntn4* **c**, *dcx* **d**, *dmnt3a* **e**, *reelin* **f**, *parvalbumin* **g**, *nestin* **h**, *rora* **i**, *pcna* **j**, *olig4* **k**, *mecp2* and **l**, *gfap*, **m** *oatp1c1*, **n** *mct8*, **o** *mct10*, **p** *lat1*, and **q** *lat2* . Relative fold change presented used log2scale with reference done on control groups. Statistics used Kruskal-Wallis tests (Medians, Whisker boxes mean and max_black dots represents values far from the rest but not outliers)), *$p < 0.05$, **$p < 0.01$, ***$p < 0.001$).

Figure S5 (Videos) (related to Figure 4) - A 360° view of the 3D brain reconstructed after imaging the brains exposed to CLARITY

a) Wild type whole brain (avi file in folder)
b) T_3 treated whole brain (avi file in folder)
c) Mix 10x treated whole brain (avi file in folder)

Figure S6 (related to Figure 4) (BEHAVIOUR)

a) Protocol (schematic) for assessing locomotor activity. NF48 tadpoles exposed for 3 days are placed one/well in a 12 well plate. Each well contains 4ml of water. Each individual is video tracked for 10 min. Every 30 seconds, light goes on or off in order to stimulate the movements.
b) Distance covered by tadpoles over 10 minutes (detail minute by minute)
c) Distances covered by tadpoles with regards to dark and light periods.
d) Mobility video (Control): Full length video of the 12 well plate subjected to mobility assay
e) Mobility video (T_3): Full length video of the 12 well plate subjected to mobility assay
f) Mobility video (Mixture 10x): Full length video of the 12 well plate subjected to mobility assay

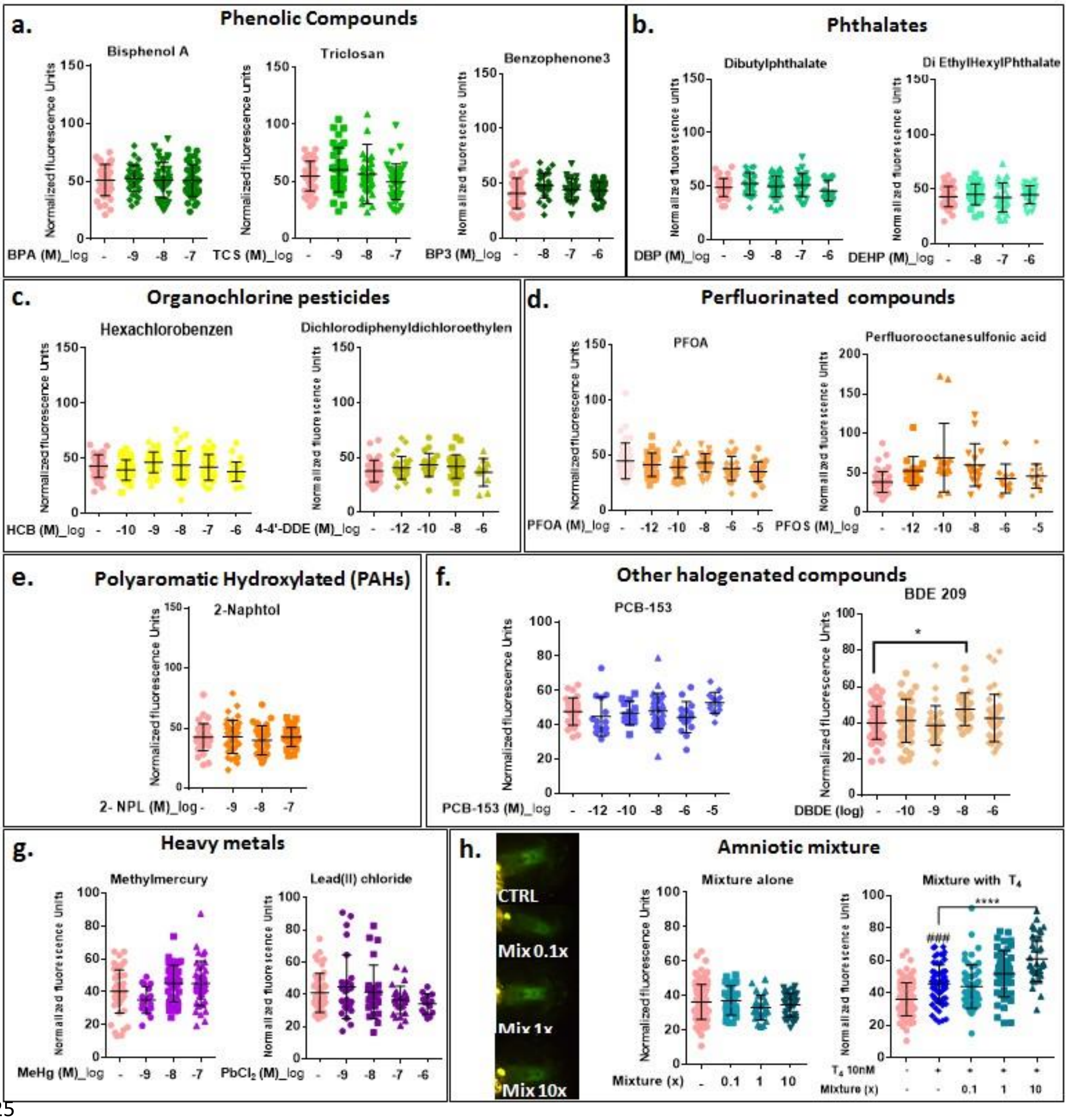
a.
Phenolic Compounds
Bisphenol A
Triclosan
Benzophenone3
Normalized fluorescence Units
BPA (M)_log - -9 -8 -7
TCS (M)_log - -9 -8 -7
BP3 (M)_log - -8 -7 -6
b.
Phthalates
Dibutylphthalate
Di EthylHexylPhthalate
DBP (M)_log - -9 -8 -7 -6
DEHP (M)_log - -8 -7 -6
c.
Organochlorine pesticides
Hexachlorobenzen
Dichlorodiphenyldichloroethylen
HCB (M)_log - -10 -9 -8 -7 -6
4-4'-DDE (M)_log - -12 -10 -8 -6
d.
Perfluorinated compounds
PFOA
Perfluorooctanesulfonic acid
PFOA (M)_log - -12 -10 -8 -6 -5
PFOS (M)_log - -12 -10 -8 -6 -5
e.
Polyaromatic Hydroxylated (PAHs)
2-Naphtol
2- NPL (M)_log - -9 -8 -7
f.
Other halogenated compounds
PCB-153
BDE 209
PCB-153 (M)_log - -12 -10 -8 -6 -5
DBDE (log) - -10 -9 -8 -6
g.
Heavy metals
Methylmercury
Lead(II) chloride
MeHg (M)_log - -9 -8 -7
PbCl2 (M)_log - -9 -8 -7 -6
h.
CTRL
Mix 0.1x
Mix 1x
Mix 10x
Amniotic mixture
Mixture alone
Mixture with T4
Mixture (x) - 0.1 1 10
T4 10nM - + + + +
Mixture (x) - - 0.1 1 10

Figure S1

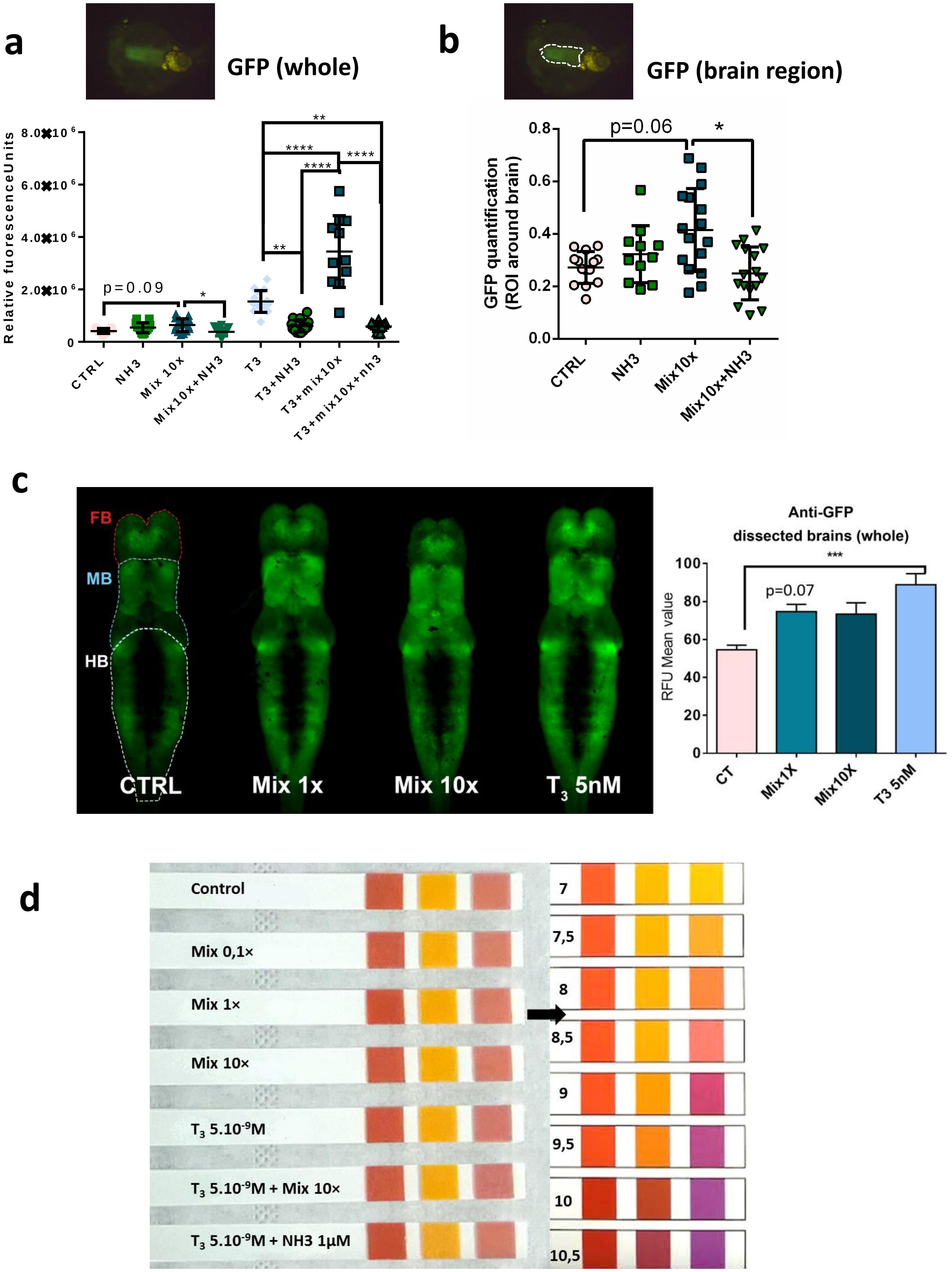
a
GFP (whole)
b
GFP (brain region)
Relative fuorescenceUnits
8.0×10^6
6.0×10^6
4.0×10^6
2.0×10^6
0
p=0.09
*
**
****
****
****
**
CTRL
NH3
Mix 10x
Mix10x+NH3
T3
T3+NH3
T3+mix10x
T3+mix10x+nh3
GFP quantification
(ROI around brain)
0.8
0.6
0.4
0.2
0.0
p=0.06
*
CTRL
NH3
Mix10x
Mix10x+NH3
c
FB
MB
HB
CTRL
Mix 1x
Mix 10x
T3 5nM
Anti-GFP
dissected brains (whole)
***
p=0.07
RFU Mean value
100
80
60
40
20
0
CT
Mix1X
Mix10X
T3 5nM
d
Control
Mix 0,1×
Mix 1×
Mix 10×
T3 5.10-9M
T3 5.10-9M + Mix 10×
T3 5.10-9M + NH3 1µM
7
7,5
8
8,5
9
9,5
10
10,5

Figure S2

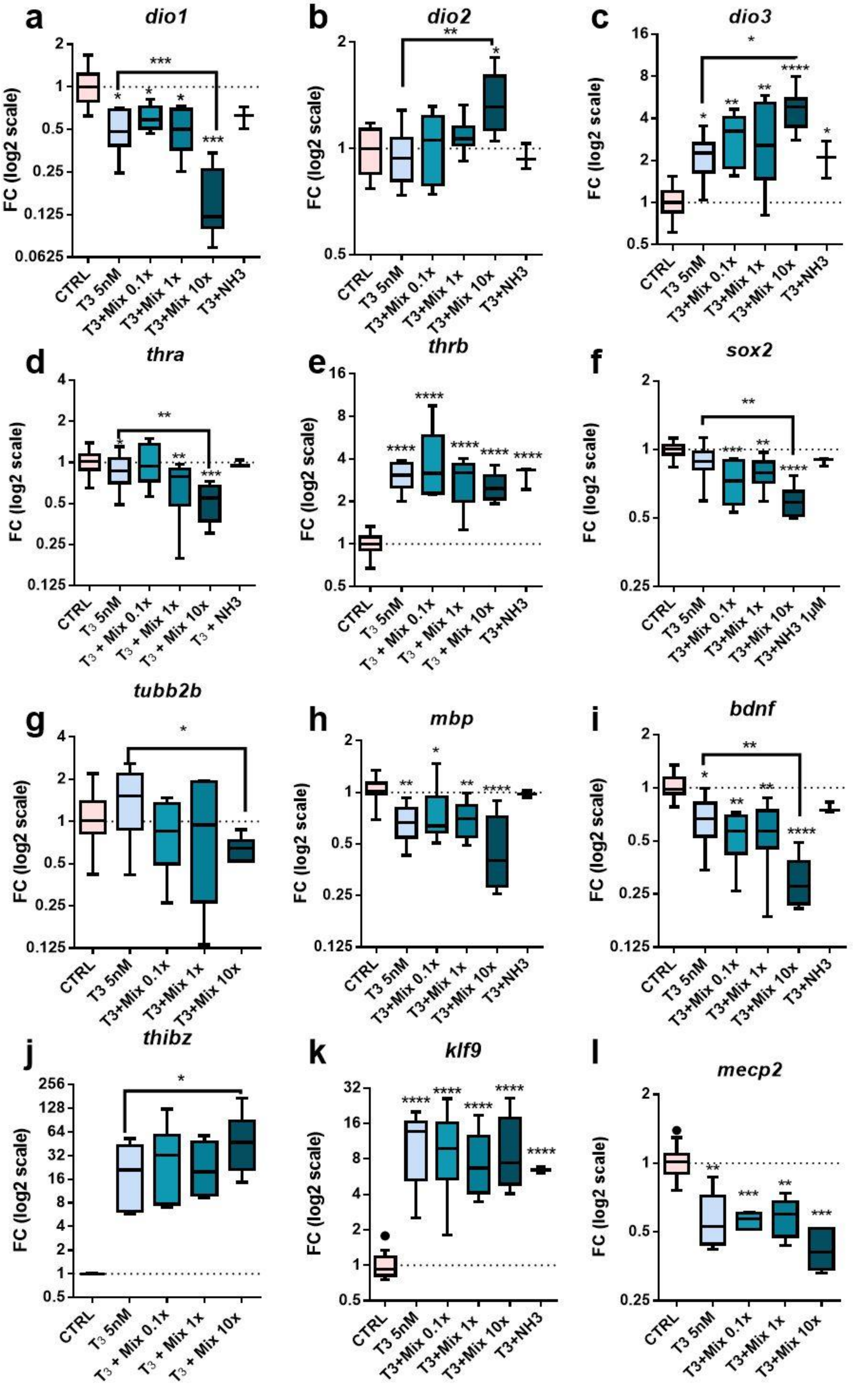
a
dio1
b
dio2
c
dio3
d
thra
e
thrb
f
sox2
g
tubb2b
h
mbp
i
bdnf
j
thibz
k
klf9
l
mecp2
FC (log2 scale)
CTRL
T3 5nM
T3+Mix 0.1x
T3+Mix 1x
T3+Mix 10x
T3+NH3
T3 + Mix 0.1x
T3 + Mix 1x
T3 + Mix 10x
T3 + NH3
T3+NH3 1µM

Figure S3

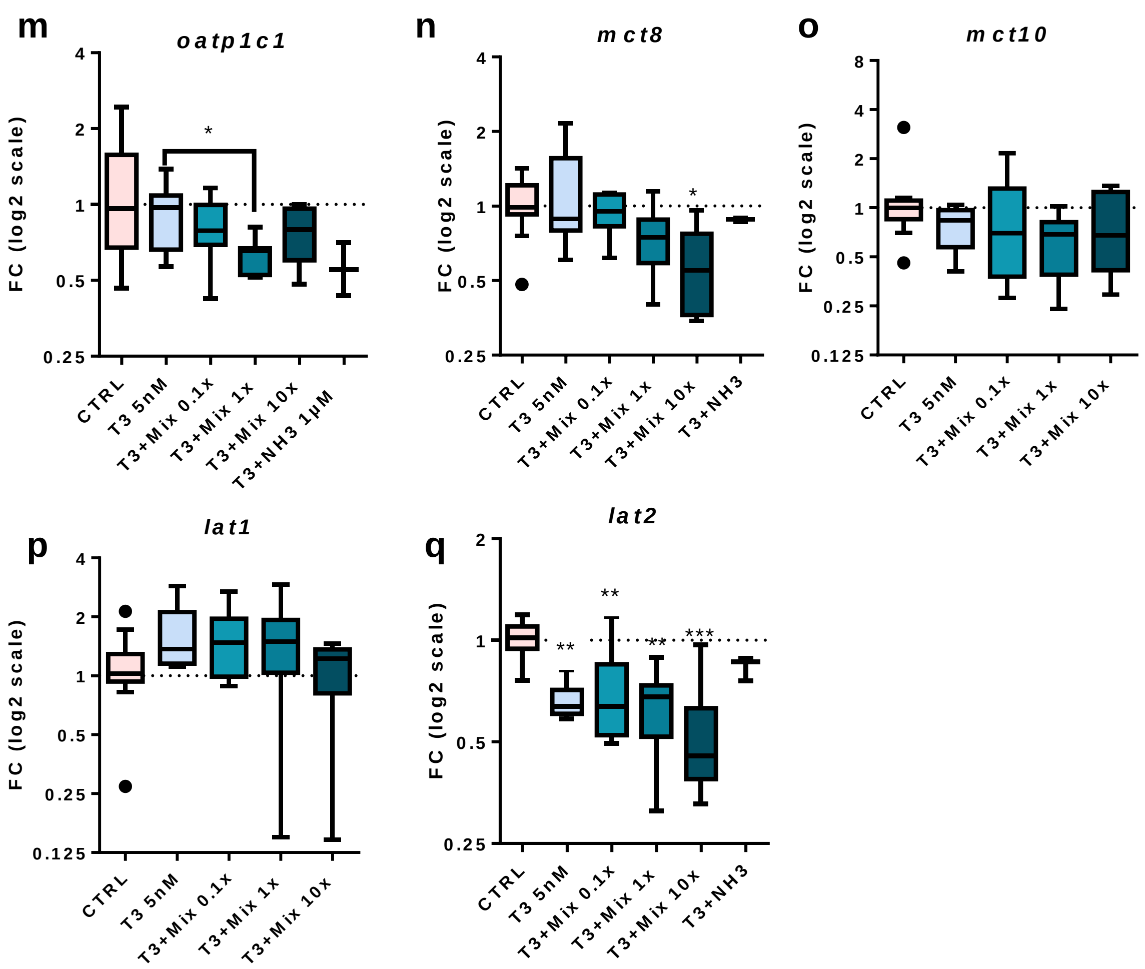

Figure S3 (continued)

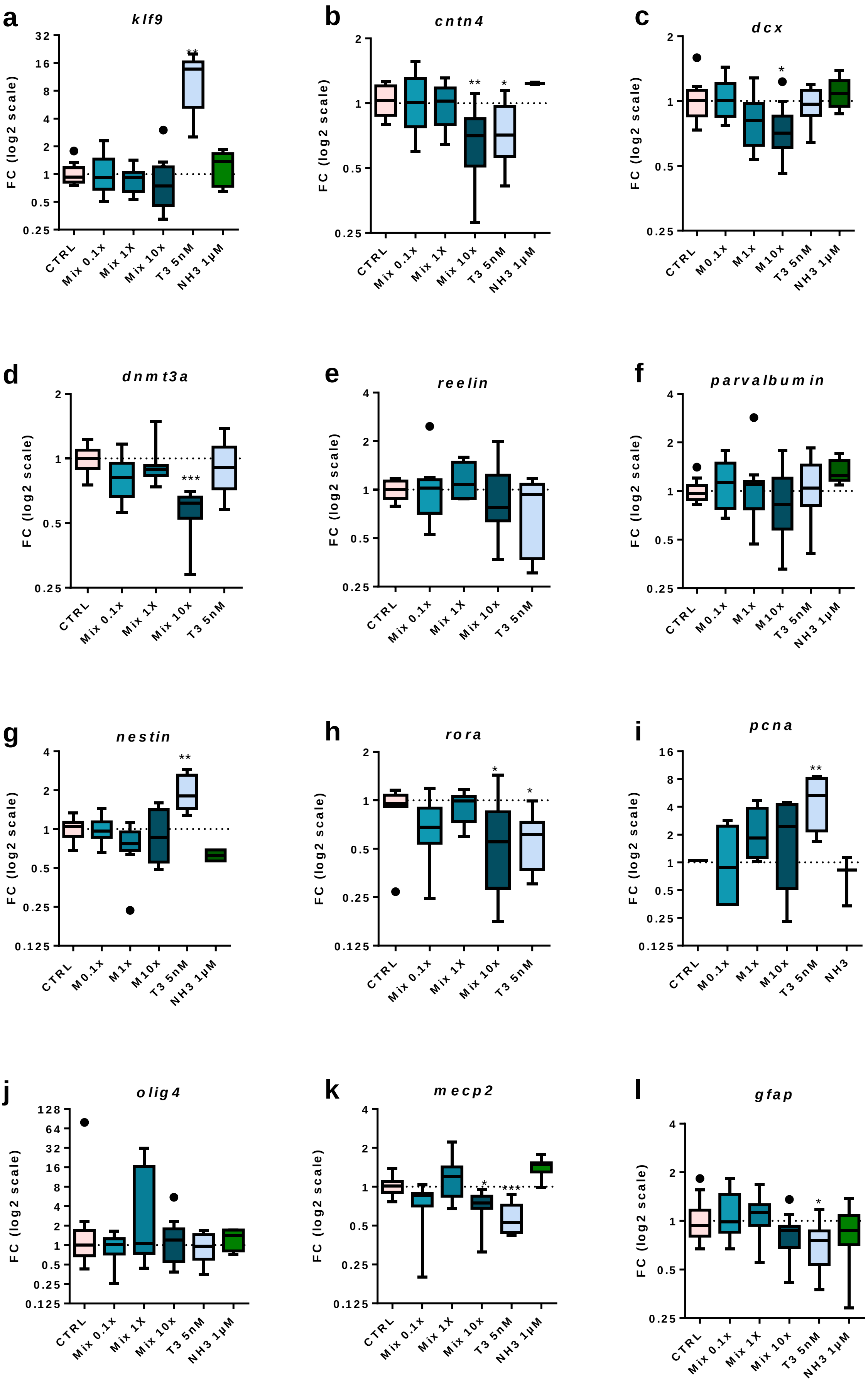

a
klf9
FC (log2 scale)
0.25
0.5
1
2
4
8
16
32
**
CTRL
Mix 0.1x
Mix 1X
Mix 10x
T3 5nM
NH3 1µM
b
cntn4
FC (log2 scale)
0.25
0.5
1
2
**
*
CTRL
Mix 0.1x
Mix 1X
Mix 10x
T3 5nM
NH3 1µM
c
dcx
FC (log2 scale)
0.25
0.5
1
2
*
CTRL
M0.1x
M1x
M10x
T3 5nM
NH3 1µM
d
dnmt3a
FC (log2 scale)
0.25
0.5
1
2
***
CTRL
Mix 0.1x
Mix 1X
Mix 10x
T3 5nM
e
reelin
FC (log2 scale)
0.25
0.5
1
2
4
CTRL
Mix 0.1x
Mix 1X
Mix 10x
T3 5nM
f
parvalbumin
FC (log2 scale)
0.25
0.5
1
2
4
CTRL
M0.1x
M1x
M10x
T3 5nM
NH3 1µM
g
nestin
FC (log2 scale)
0.125
0.25
0.5
1
2
4
**
CTRL
M0.1x
M1x
M10x
T3 5nM
NH3 1µM
h
rora
FC (log2 scale)
0.125
0.25
0.5
1
2
*
*
CTRL
Mix 0.1x
Mix 1X
Mix 10x
T3 5nM
i
pcna
FC (log2 scale)
0.125
0.25
0.5
1
2
4
8
16
**
CTRL
M0.1x
M1x
M10x
T3 5nM
NH3
j
olig4
FC (log2 scale)
0.125
0.25
0.5
1
2
4
8
16
32
64
128
CTRL
Mix 0.1x
Mix 1X
Mix 10x
T3 5nM
NH3 1µM
k
mecp2
FC (log2 scale)
0.125
0.25
0.5
1
2
4
*
***
CTRL
Mix 0.1x
Mix 1X
Mix 10x
T3 5nM
NH3 1µM
l
gfap
FC (log2 scale)
0.25
0.5
1
2
4
*
CTRL
Mix 0.1x
Mix 1X
Mix 10x
T3 5nM
NH3 1µM

Figure S4

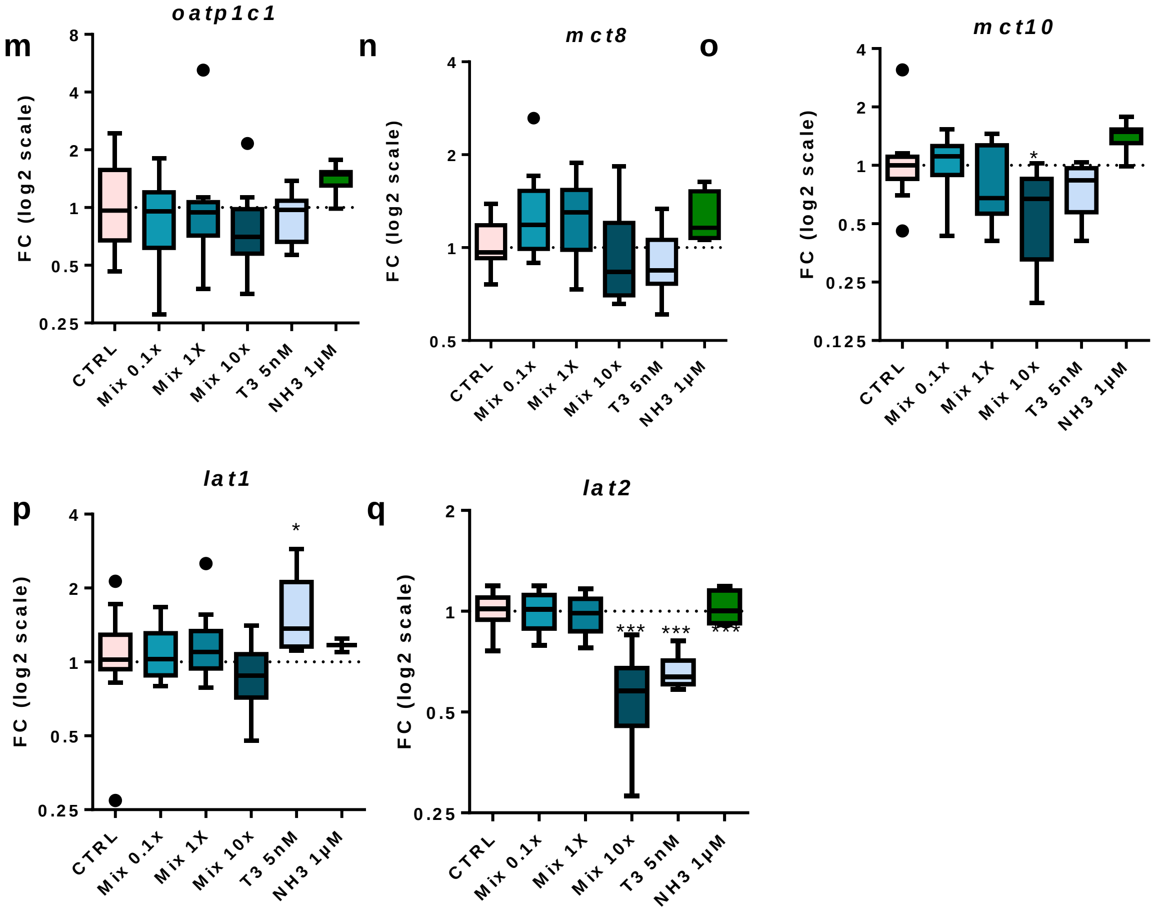

Figure S4 (continued)

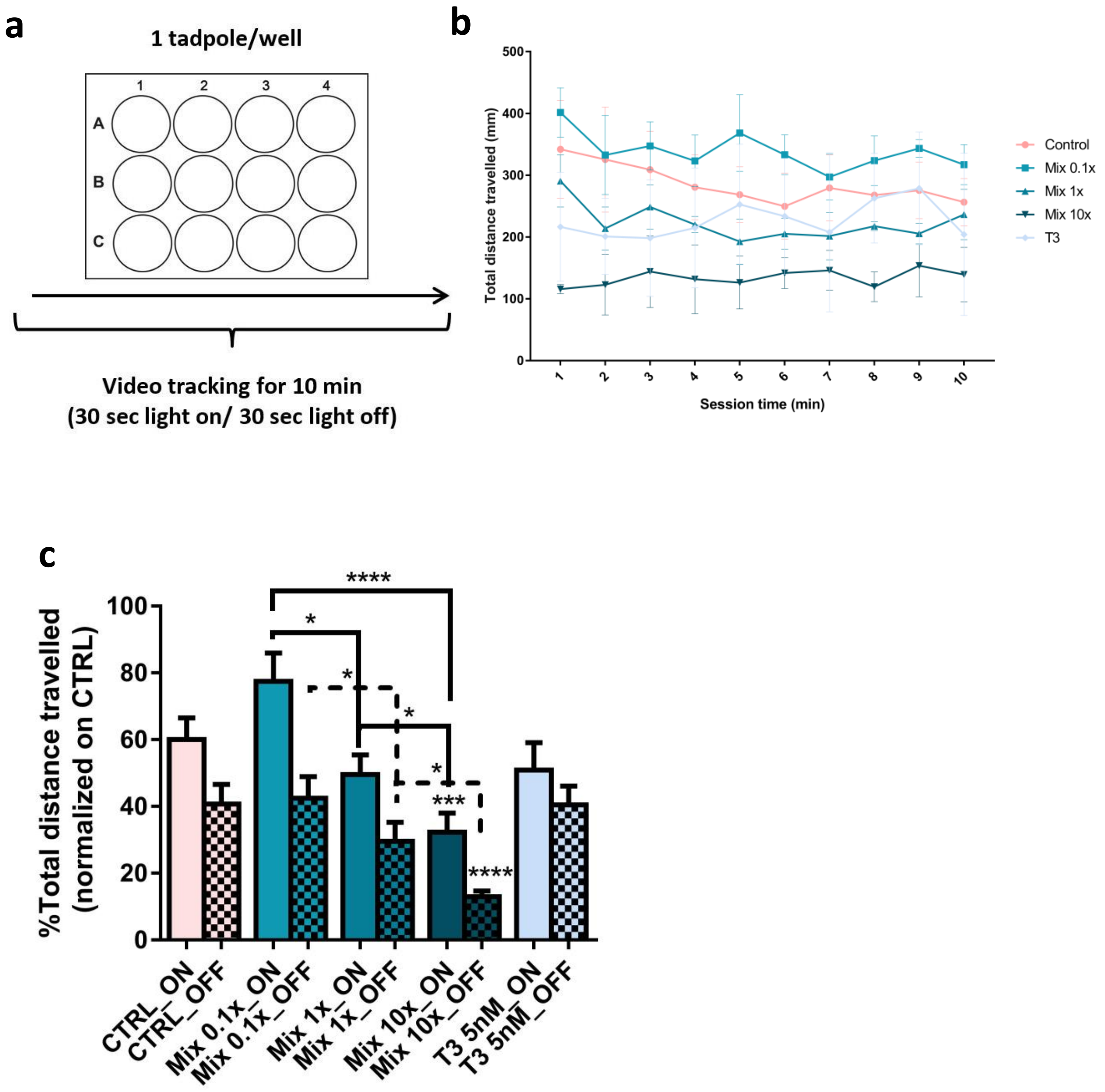
a
1 tadpole/well
1
2
3
4
A
B
C
Video tracking for 10 min
(30 sec light on/ 30 sec light off)
b
Total distance travelled (mm)
Session time (min)
Control
Mix 0.1x
Mix 1x
Mix 10x
T3
c
%Total distance travelled
(normalized on CTRL)
CTRL_ON
CTRL_OFF
Mix 0.1x_ON
Mix 0.1x_OFF
Mix 1x_ON
Mix 1x_OFF
Mix 10x_ON
Mix 10x_OFF
T3 5nM_ON
T3 5nM_OFF

Figure S6

Gene name	Forward Sequence (5' - 3')	Reverse Sequence (5' - 3')	Gene Accession/Xenbase id
**dio1**	5'-CAGCAGATGAATGGGCATTGA-3'	5'-TGTCTAACACTACTGGGCAAGAAGGT-3'	
NM_001095667.1			NM_001095667.1
Long Copy			XB-GENE-979948
Short copy			XB-GENE-17345940
**dio2**	5'-CAAATCCAGCCAGAGAGTCAATGG-3'	5'-TGACCTGCTTGTAGGCATCCA-3'	
AF354707.1			AF354707.1
Long Copy			XB-GENE-1017482
Short copy			XB-GENE-17339189
**dio3**	5'-CACAAAAAGTGCGACCAAACG-3'	5'-GCCTTGTTGCAGTTTACT-3'	
NM_001087863.1			NM_001087863.1
Long Copy			XB-GENE-865707
Short Copy			XB-GENE-17336015
**thra**	5'-CGCCTTGGTCTCTTCGGAT-3'	5'-CCAGAAGTGGGGAATGTTGTGTT-3'	
NM_001088126.1			NM_001088126.1
Long Copy			XB-GENE-5966552
Short Copy			XB-GENE-17340389
**thrb**	5'-AAGAGTGGTTGATTTTGCCAAAA-3'	5'-AGGGACATGATCTCCATACAACAG-3'	
NM_001097064.1			NM_001097064.1
Long Copy			XB-GENE-6070716
Short Copy			XB-GENE-6252616
sox2	5'-CCAGTCCACCTGTAGTCACCTCT-3'	5'-CACTTCTGCCCCCAGGTAGGTAC-3'	
NM_001088222.1			NM_001088222.1
AF022928.1			AF022928.1
Long Copy			XB-GENE-865099
Short Copy			XB-GENE-17341038
tubb2b (ntub)	5'-ACACGGCATTGATCCTACAG-3'	5'-AGCTCCTTCGGTGTAATGAC-3'	
NM_001086064.1			NM_001086064.1
Long Copy			XB-GENE-866032
Short Copy			XB-GENE-17340462

mpb	5'-GATCAAAGCAAATGGCAACA-3'	5'-AACCTGCCCAGTGAGTCAAG-3'	
NM_001090291.1			NM_001090291.1
Long Copy			XB-GENE-959622
Short Copy			XB-GENE-17333093
bdnf	5'-CCCCCATGAAAGAAGCCAGT-3'	5'-ACTGGGACCACCTATGCTCT-3'	
NM_001085482.1			NM_001085482.1
Long Copy			XB-GENE-6252642
Short Copy			XB-GENE-955559
thibz	5'-ACCTCCACAGAATCAGCAGC-3'	5'-GCAGAGAACGAGCAAGGAGT-3'	
NM_001085805.1			NM_001085805.1
Long Copy			XB-GENE-6252652
Short Copy			XB-GENE-865112
klf9	5'-TGTGGCAAAGTTTATGGGAAGTCT-3'	5'-GGCGTTCACCTGTATGGACTCT-3'	
NM_001085597.1			NM_001085597.1
U35409.1			U35409.1
Long Copy			XB-GENE-865026
Short Copy			XB-GENE-6252653
mecp2	5'-CCATGTACGAAGACCCCACTCT-3'	5'-GACCAGATTTTCTTTGCTTGAGTTT-3'	
NM_001088385.1			NM_001088385.1
Long Copy			XB-GENE-17346751
Short Copy			XB-GENE-494744
cntn4	5'-CAGAGCAAAGGACGCCTACA-3'	5'-AGCACTGATACATGCCGGAG-3'	
NM_001091581.1			NM_001091581.1
Long Copy			XB-GENE-17337426
Short Copy			XB-GENE-866211
dcx	5'-ACACCCAAATCCAAGCAGTC-3'	5'-TCAGAGTCATCCAGAGACAGG-3'	
No NCBI			
Long Copy			XB-GENE-17344744
Short Copy			XB-GENE-17344745

dnmt3a	5'-CCAGAAAGGTTTTCCCATCA-3'	5'-CGATCATCTGCTTGTTCTGG-3'	
No NCBI			
Long Copy			XB-GENE-6488573
Short Copy			XB-GENE-17330625
reelin	5'-TTGTGAATATTCTTTTGCCTGCTAGT-3'	5'-GCCACCAGCGGAAACG-3'	
AF427525.1			AF427525.1
Long Copy			XB-GENE-982846
Short Copy			XB-GENE-17331849
parvalbumin	5'-AGCCGCTGACTCATTTAACC-3'	5'-ACTTCGATCCTGGTCGAGAA-3'	Sequence in the box
No NCBI			
Long Copy			Xelaev18023736m
Short Copy			Xelaev18025727m
nestin	5'-GAGGTGGCCACATACAGGTC-3'	5'-TGCAGTTCATTTTTGTCACCA-3'	
NM_001087857.1			NM_001087857.1
Long Copy			XB-GENE-17340193
Short Copy			XB-GENE-865974
Rora	5'-TGTAAGGGTTTCTTCCGACGAA-3'	5'-TCTGGCGTGGACATGAGTAAGT-3'	Sequence in the box
No NCBI			
Long Copy			XB-GENE-17343726
Short Copy			XB-GENE-17343727
pcna	5'-CGTCGCGGTAATCCCTTACA-3'	5'-CCAACACCTTCTTCAGGATGGA-3'	
NM_001087542.1			NM_001087542.1
Long Copy			XB-GENE-972527
Short Copy			XB-GENE-17342178
olig4	5'-GCGGGAAGTGATGCCATACT-3'	5'-TGGAAATTTTGGAGAGCTTTCG-3'	
No NCBI			
Long Copy			XB-GENE-6488436
Short Copy			XB-GENE-17335240

mct8	5'-CATTGCAGGCCTCTTACGTG-3'	5'-TCTCTCATGGACCAGCGGTA-3'	
XM_018232946.1			XM_018232946.1
Long Copy			XB-GENE-17342332
Short Copy			XB-GENE-17342333
oatp1c1	5'-GTCCGTACTGCTGCTCGATGT-3'	5'-TGGGAGCGTCTTCCAGTACAA-3'	
No NCBI			
Long Copy			XB-GENE-17343436
Short Copy			XB-GENE-17343437
mct10	5'-TTGGTCCACCTATTGCAGGTATT-3'	5'-TGGATCCATGGTATCAGGCATA-3'	
No NCBI			
Long Copy			XB-GENE-6487093
Short Copy			XB-GENE-17345674
lat1	5'-GGCCTATCAAGGTAAACATTTTGC-3'	5'-CACACTCAACAGGTGTCATGTAGAAT-3'	
NM_001090065.1			NM_001090065.1/NM_001096373.1
Long Copy			XB-GENE-6254908
Short Copy			XB-GENE-865334

	Family/ complet name	Molecule (abbreviation)_CAS n°	Use	DETECTION RATE in pregnant women (non pregnant) WOODRUFF 2011	Tolerable daily intake	Concentrations measured in urine during pregnancy	Concentrations measured in human serum	Median concentration found in umbilical cord blood serum	Median concentration measured in amniotic fluid	Molecular Weight	Molarity/ mass	Final Concentration in the mixture 0.1x	Final concentration in the mixture 1X	Final concentraion in the mixture 10X	Mixture mother solution concentrations (100 000x in DMSO)	Volume of each compound at 10-1M concentration (µl)
1	Environmental phenols	Bisphenol A (BPA)_80-05-7	plasticizer	96% (96%)	4 µg/kg bw/day	1.63 µg/L (Woodruff et al., 2011)_1,36µg/l (Aris 2014)_0.4µg/L (Jiménez-Díaz et al, 2016)	0,49+/-2,47 ng/ml (Burstyn et al, 2013) (pregnant mother)_7.43 µg/L (Shekhar et al, 2016)	1,23 ng/ml (Aris 2014)_0,16ng/ml (Gerona et al 2013)	[0-0,46] (Eldow et al;, 2012)_second trimester: [nd-0.75ng/ml) median 0.47 ng/ml (Chen et al;, 2011)_ ND (Philippat et al;, 2013)_ 7.75 µg/L (Shekhar et al, 2016)	228.29 g/mol	0.2 10-8M (0.5 µg/L	0.2 10-9M	0.2 10-8M	0.2 10-7M	0.2 10-3M	2
2	Environmental phenols	Triclosan (TCS), dichlorophenoxyphenol _3380-34-5	antibacterian concervator (cosmetic)	87%(81%)	Substances for which an Acceptable Daily Intake or Tolerable Daily Intake could not be established, but where the present use could be accepted.5 mg/kg	23.81µg/l (Woodruff et al., 2011)			19.4µg/l (amniotioc high quartile) Philippat et al., 2013_7.04 µg/L (Shekhar et al, 2016)	289.54 g/mol	0.7 10-7M (19.4µg/l)	0.7 10-8M	0.7 10-7M	0.7 10-6M	0.7 10-2M	70
3	Environmental phenols	benzophenone-3 (BP-3), oxybenzone_131-57-7	UV filter (cosmetics)_	100%(98%)	0.03mg/kg b.w./day	38.09 µg/l (Woodruff et al., 2011) _4.7µg/L Median/ Max : 3200µg/L (Hines et al, 2015)__77µg/L Median (Phillipat et al, 2013)			0-15.7µg/l (amniotic) median 0,8 µg/l Philippat et al., 2013	182.22 g/mol	0.86 10-7M (15.7 µg/l)	0.9 10-8M	0.86 10-7M	0.86 10-6M	0.86 10-2M	86
8	Phthalates	Dibutyl phthalate (DBP)_ 84-74-2	softener (plastic, shampoo)	99% (99%)_MBP	0.01 mg/kg body weight per day	18.83 µg/l (geometric mean in pregnant women 50th percentile 17.1 µg/l 95th percentile 143.8 µg/l (Woodruff et al;, 2011) data for MBP _____metabolite MBP in mother urine 1st trimester [8,9;26,9;78;30,9] max 541 mg/l (females group) _ [19,4;28,1;79,6;232,6] max=524 mg/l (males group) Huang e al *; 2009		68.14 µg/l (cord blood) (Huang et al. 2014)__cord blood serum 6µg/l (Zhang et al., 2009)._ Meconium 6mg/g (Zhang et al., 2009)	around 80µg/ of MBP in amniotic fluid (50th percentile male et females) [39.3;45.6;85.5;134.6] max =192 µg/l (female groupe for MBP) Huang et al 2009 [28,4;44,3;81,3;127,8] max=145 µ/L ml male group Huang et al 2009	278.34 g/mol MBP 222.24g/mol	0.24 10-6M (53.4 µg/l)	0.24 10-7M	0.24 10-6M	0.24 10-5M	0.24 10-1M	240
7	Phthalates	di-2-éthylhexylphthalate (DEHP)_117-81-7	plastic softener	100% (100%)_MEP	0.05 mg/kg body weight per day	226.53 µg/l (geometric mean) (Woodrufff at al 2011)		187.16 µg/l (cord blood) Huang et al., 2014__cord blood serum 1µg/l (Zhang et al., 2009)_ Meconium 3mg/g (Zhang et al., 2009)_mothermilk 10µg/l (Main et al 2006)	DEHP metabolite at 0.27µg/l (median Jensen et al 2012)_ 22.1 µg/l 50 percentile (amniotic fluid MEHP)[0;5;24;91,1] (max 148µg/l) for females and [0;2,6;22,1;100,6] max 110 µg/l males (amnitoic fluid MEHP) Huang et al., 2009_1.7-2.6 µg/l Silva et al. 2004 (metab)__	390.56 g/mol MEHP: 278.15	0.08 10-6M (22.1 µg/l)	0.1 10-7M	0.1 10-6M	0.1 10-5M	0.11 10-1M	110
5	Organochlorine pesticides	Hexachlorobenzen (HCB)_118-74-1	fungicide, pesticide and precursor of synthetic caoutchouc	100% (99%)	0.16 mg/kg body weight per day		13.74 ng/g lipid (Woodruff et al., 2011) (pregnant mother)		0.023µg/l (median levels) or 0.097µg/l (mean) in amniotic fluids 100 women tenerife (Luzardo et al, 2009)__0.24 ng/l (Germany Van der Ven et al. 1992)__0.02 ng/l (mean in Tanzania Van der Ven et al. 1992)__0.032 µg/l (mean in Meeker et al 2009)__6.6µg/l (mean in Daglioglu et al. 2013)	284.78 g/mol	0.8 10-11M (0.0023µg/l)	0.8 10-12M	0.8 10-11M	0.8 10-10M	0.8 10-6M	0.8
4	Organochlorine pesticides	Dichlorodiphenyldichlor oethylene (4-4' DDE, p-p'DDE metabolite DDT), _72-55-9	insecticide (DDT metabolite)	100% (99%)	PTDI of 0.01 mg/kg b.w (provisional TDI)		198.34 ng/g lipid (Woodruff et al., 2011) (pregnant mother)		0.21 µg/l mean (Foster et al., 2000)__0.030µg/l (mean in Luzardo et al. 2009)__0.384µg/l (mean Meeker at l 2009)__5.4µg/l mean (Daglioglu et al. 2013)	318.02 g/mol	0.66 10-9M (0.21 µg/l)	0.7 10-10M	0.66 10-9M	0.66 10-8M	0.66 10-4M	0.66
13	Perfluorinated compounds	Perfluorooctanoic acid (PFOA)_ 335-67-1	fluorosurfactant	99% (99%)	150 ng/kg b.w/day		50th percentile: 2.6µg/l; 95th percentile 5.6µg/l (Woodruff et al., 2011) _[0.309-7.31] median 1.045 µg/l (Cariou et al., 2015) (pregnant mother)	[0.311-7.06] µg/l median 0.860 µg/l (Cariou et al. 2015)	0.1-1.8 µg/l mean 0.3µg/l (amniotic fluid) Stei et al., 2012	414.07 g/mol	0.43 10-8M (1.8µg/l)	0.43 10-9M	0.43 10-8M	0.43 10-7M	0.43 10-3M	4.3
12	Perfluorinated compounds	Perfluorrooctanyl sulfonate (PFOS)_ 1763-23-1	fluorosurfactant	99% (100%)	0.3 µg/kg bw/day		50th percentile: 12µg/l; 95th percentile 21.8µg/l (Woodruff et al., 2011) __[0.316-24..5] median 3.065 µg/l (Cariou et al; 2015) (pregnant mother)	[nd-8.04]µg/l median 1.115µg/l (Cariou et al., 2015)	1.1-4.5 µg/L (amniotic fluid) Jensen et al;, 2012	538.23 g/mol	0.8 10-8M (4.5µg/l)	0.8 10-9M	0.8 10-8M	0.8 10-7M	0.8 10-3M	8
11	Poly aromaric Hydroxylated compounds (PAHs)	2-Napthol_ 135-19-3	fluorescent colorless crystallin solid (naphtalen derivative)	100%(100%)	none	2.49µg/l in pregnant women (geometric mean) (50th percentile: 2.4µg/l; 95th percentile 14.7µg/l (Woodruff et al., 2011)			0.72µg/L median (amniotic fluid) Bradman et al;, 2003	144.17 g/mol	0.5 10-8M (0.72µg/l)	0.5 10-9M	0.5 10-8M	0.5 10-7M	0.5 10-3M	5
6	Poly chlorinated	Sodium perchlorate Monohydrate (NaClO4-)_ 7791-07-3	explosive properties used in aeronautics	100%(100%)	Perchlorate : 0.3 µg/kg b.w	4.3 µg/l (50th percentile:; 95th percentile 34µg/l (Woodruff et al., 2011) 3.35µg/L (maternal urine),	[-; 0,893] mean 0,417 µg/l (Blount et al., 2009) (pregnant mother)	[-; 0,480] mean 0,246 µg/l (Blount et al., 2009) _mean 0,440 µg/l (Amitai et al., 2007)	0.057–0.38 µg/l (amniotic fluid) Blount et al., 2009	122.44 g/mol	0.3 10-8M (0.38µg/l)	0.3 10-9M	0.3 10-8M	0.310-7M	0.3 10-3M	7.5 µl (à partir de 1g/L
9	Polybrominated compounds (PBDEs)	Decabromodiphenylether (BDE 209)_ 1163-19-5	flame retardant	87% (68%)	1,7µg/kg b.w	0.08–106.49µg/g creatinine (Ho & al, 2015)	1.59 ng/g lipid weight (Ho & al, 2015)	15.8 ± 9.88 ng/g− 1 lipid in placenta, 13.2 ± 7.64 ng/g− 1 lipid in breast milk, 16.5 ± 19.5 ng/g− 1 lipid in fetal cord blood, and 1.80 ± 1.99 ng/ml− 1 in neonatal urine. (Chen & al, 2014)	3795 pg/ml (all PBDE) 16% de BDE209 so: 607.2pg/ml ou ng/L Miller et al 2012	959.22 g/mol	0.63 10-9M (607.2 ng/l)	0.63 10-10M	0.63 10-9M	0.63 10-8M	0.63 10-4M	0.63
10	Polychlorinated compounds (PCBs)	2,2',4,4',5,5'-Hexachlorbiphenyl (PCB-153)_35065-27-1	flame retardant found in Arochlor (PCB mixture) (banned)	100% (100%)	20 ng/kg b.w/day		50th percentile: 8.8µg/l lipid; 95th percentile 22.5 µg/g lipid (Woodruff et al., 2011) Danish mother : 1,303µg/l (median) or 152 ng/g lipid in serum (Kristensen et al, 2016)	0.97 µg/l Portugal (cord blood) Lopes et al 2014 _0.04ng/ml (wet) cord blood 0.21µg/l German (Bucholski et al., 1996) 0.38µg/l Slovakia (Park et al., 2008) 0.21µg/l Spain (Grimalt et al., 2010)	0.009µg/l (Luzardo & al, 2009) __0.064µg/l (Meeker et al., 2009)__0.16µg/l (in Germany Van der Ven et al., 1992__0.02µg/l (in Tanzania Van der Ven et al., 1992) __ 3.8µ/l (Daglioglu et al., 2013)	360.88g/mol	0.2 10-8M (0.72µg/)	0.2 10-9M	0.2 10-8M	0.2 10-7M	0.2 10-3M	2
14	Heavy metals	Methyl mercury (MeHg cL) _115-09-3		89% (92%)	1.3 µg/kg b.w/week (TWI not per day) 0.035 to 0.08µg/kg/day		63µg/L (Obiri et al, 2016) -Ghana		0.2 µg/ l max 11µg/l (amniotic fluid) mercury Kozikowska et al., 2013__0.37 µg/l (Pier Franca Luglie et a 2005)	215.62 g/mol	0.5 10-7M (11 µg/l)	0.5 10-8M	0.5 10-7M	0.5 10-6M	0.5 10-2M	50
15	Heavy metals	Lead (II) chloride (PbCl2)_ 7758-95-4	pigments, fuels	94% (99%)	ADI (acceptable daily intake) 7µg/kg/day	Median : 0.5µg/L (Christensen, 2012)	1.290 µg/dL ±0.578 (Baranowska-Bosiack et al, 2016)(Pregnant mothers)<br><br>5th-95th percentile(Nunes et al, 2010) en µg/L<br>8.0–47.0 German<br>5.0–132.0 UK<br>39.7–275.7 Italian<br>11.4–62.8 French<br>3.5–170.0 Swedish<br>5.1–163.0 Brazil<br>58µg/L (Obiri&al, 2016) -Ghana	30µg/l (Semczuk et al.,1998)__24µg/l (Senczuk et al 1994)__in breast milk : 0.174 µg/dL ±1.15 (Baranowska-Bosiack et al, 2016)	59.6 µg/l Korpela et al., 1986	278.108 g/mol	0.21 10-6M (59.6µg/l)	0.21 10-9M	0.21 10-8M	0.21 10-7M	0.21 10-3M	2.1

Supplementary References related to Supplementary Table 1

Amitai, Y., et al. (2007), 'Gestational exposure to high perchlorate concentrations in drinking water and neonatal thyroxine levels', *Thyroid,* 17 (9), 843-50.

Aris, A. (2014), 'Estimation of bisphenol A (BPA) concentrations in pregnant women, fetuses and nonpregnant women in Eastern Townships of Canada', *Reprod Toxicol,* 45, 8-13.

Baranowska-Bosiacka, I., et al. (2016), 'Environmental Lead (Pb) Exposure Versus Fatty Acid Content in Blood and Milk of the Mother and in the Blood of Newborn Children', *Biol Trace Elem Res,* 170 (2), 279-87.

Blount, B. C., et al. (2009), 'Perinatal exposure to perchlorate. thiocyanate, and nitrate in New Jersey mothers and newborns', *Environ Sci Technol,* 43 (19), 7543-9.

Bradman, A., et al. (2003), 'Measurement of pesticides and other toxicants in amniotic fluid as a potential biomarker of prenatal exposure: a validation study', *Environ Health Perspect,* 111 (14), 1779-82.

Burstyn, I., et al. (2013), 'Maternal exposure to bisphenol-A and fetal growth restriction: a case-referent study', *Int J Environ Res Public Health,* 10 (12), 7001-14.

Cariou, R., et al. (2015), 'Perfluoroalkyl acid (PFAA) levels and profiles in breast milk, maternal and cord serum of French women and their newborns', *Environ Int,* 84, 71-81.

Chen, M., et al. (2011), 'Determination of bisphenol-A levels in human amniotic fluid samples by liquid chromatography coupled with mass spectrometry', *J Sep Sci,* 34 (14), 1648-55.

Chen, Z. J., et al. (2014), 'Polybrominated diphenyl ethers (PBDEs) in human samples of mother-newborn pairs in South China and their placental transfer characteristics', *Environ Int,* 73, 77-84.

--- (2014), 'Polybrominated diphenyl ethers (PBDEs) in human samples of mother-newborn pairs in South China and their placental transfer characteristics', *Environ Int,* 73, 77-84.

Christensen, K. L. Y. (2013), 'Metals in blood and urine, and thyroid function among adults in the United States 2007-2008', *International Journal of Hygiene and Environmental Health,* 216 (6), 624-32.

Daglioglu, N., et al. (2013), 'Polychlorinated biphenyls and organochlorine pesticides in amniotic fluids of pregnant women in south-central Turkey', *Toxicological and Environmental Chemistry,* 95 (6), 954-61.

Edlow, A. G., et al. (2012), 'Fetal bisphenol A exposure: concentration of conjugated and unconjugated bisphenol A in amniotic fluid in the second and third trimesters', *Reprod Toxicol,* 34 (1), 1-7.

Foster, W., et al. (2000), 'Detection of endocrine disrupting chemicals in samples of second trimester human amniotic fluid', *J Clin Endocrinol Metab,* 85 (8), 2954-7.

Gerona, R. R., et al. (2013), 'Bisphenol-A (BPA), BPA glucuronide, and BPA sulfate in midgestation umbilical cord serum in a northern and central California population', *Environ Sci Technol,* 47 (21), 12477-85.

Grimalt, J. O., et al. (2010), 'An evaluation of the sexual differences in the accumulation of organochlorine compounds in children at birth and at the age of 4 years', *Environ Res,* 110 (3), 244-50.

Hines, E. P., et al. (2015), 'Concentrations of environmental phenols and parabens in milk, urine and serum of lactating North Carolina women', *Reprod Toxicol,* 54, 120-8.

Hinwood, A. L., et al. (2013), 'Cadmium, lead and mercury exposure in non smoking pregnant women', *Environ Res,* 126, 118-24.

Ho, K. L., et al. (2015), 'Urinary bromophenol glucuronide and sulfate conjugates: Potential human exposure molecular markers for polybrominated diphenyl ethers', *Chemosphere,* 133, 6-12.

Huang, P. C., et al. (2009), 'Association between prenatal exposure to phthalates and the health of newborns', *Environ Int,* 35 (1), 14-20.

Huang, Y., et al. (2014), 'Phthalate levels in cord blood are associated with preterm delivery and fetal growth parameters in Chinese women', *PLoS One,* 9 (2), e87430.

Iwai-Shimada, M., et al. (2015), 'Methylmercury in the breast milk of Japanese mothers and lactational exposure of their infants', *Chemosphere,* 126, 67-72.

Jensen, M. S., et al. (2012), 'Phthalates and perfluorooctanesulfonic acid in human amniotic fluid: temporal trends and timing of amniocentesis in pregnancy', *Environ Health Perspect,* 120 (6), 897-903.

Jimenez-Diaz, I., et al. (2016), 'Urinary levels of bisphenol A, benzophenones and parabens in Tunisian women: A pilot study', *Sci Total Environ,* 562, 81-8.

Kim, J. T., et al. (2015), 'Partitioning behavior of heavy metals and persistent organic pollutants among feto-maternal bloods and tissues', *Environ Sci Technol,* 49 (12), 7411-22.

Korpela, H., et al. (1986), 'Lead and cadmium concentrations in maternal and umbilical cord blood, amniotic fluid, placenta, and amniotic membranes', *Am J Obstet Gynecol,* 155 (5), 1086-9.

Kozikowska, I., et al. (2013), 'Mercury concentrations in human placenta, umbilical cord, cord blood and amniotic fluid and their relations with body parameters of newborns', *Environ Pollut,* 182, 256-62.
Kristensen, S. L., et al. (2016), 'Prenatal exposure to persistent organochlorine pollutants and female reproductive function in young adulthood', *Environ Int,* 92-93, 366-72.
LeFeng, W., et al. (2015), 'Association of urinary metal profiles with altered glucose levels and diabetes risk: a population-based study in China', *PLoS One,* 10 (4), e0123742.
Li, Y. F., et al. (2008), 'Mercury in human hair and blood samples from people living in Wanshan mercury mine area, Guizhou, China: an XAS study', *J Inorg Biochem,* 102 (3), 500-6.
Lopez-Espinosa, M. J., et al. (2010), 'Prenatal exposure to organochlorine compounds and neonatal thyroid stimulating hormone levels', *J Expo Sci Environ Epidemiol,* 20 (7), 579-88.
Luglie, P. F., et al. (2005), 'Effect of amalgam fillings on the mercury concentration in human amniotic fluid', *Arch Gynecol Obstet,* 271 (2), 138-42.
Luzardo, O. P., et al. (2009), 'Determinants of organochlorine levels detectable in the amniotic fluid of women from Tenerife Island (Canary Islands, Spain)', *Environ Res,* 109 (5), 607-13.
McKean, S. J., et al. (2015), 'Prenatal mercury exposure, autism, and developmental delay, using pharmacokinetic combination of newborn blood concentrations and questionnaire data: a case control study', *Environ Health,* 14, 62.
Meeker, J. D., et al. (2009), 'Serum and follicular fluid organochlorine concentrations among women undergoing assisted reproduction technologies', *Environ Health,* 8, 32.
Mohr, S., et al. (2015), 'Polychlorinated biphenyls in umbilical cord serum of newborns from Rio Grande do Sul state, Brazil', *Clin Chim Acta,* 451 (Pt B), 323-8.
Nunes, J. A., et al. (2010), 'A simple method based on ICP-MS for estimation of background levels of arsenic, cadmium, copper, manganese, nickel, lead, and selenium in blood of the Brazilian population', *J Toxicol Environ Health A,* 73 (13-14), 878-87.
Obiri, S., et al. (2016), 'Levels of arsenic, mercury, cadmium, copper, lead, zinc and manganese in serum and whole blood of resident adults from mining and non-mining communities in Ghana', *Environ Sci Pollut Res Int*.
Park, J. S., et al. (2008), 'Placental transfer of polychlorinated biphenyls, their hydroxylated metabolites and pentachlorophenol in pregnant women from eastern Slovakia', *Chemosphere,* 70 (9), 1676-84.
Philippat, C., et al. (2013), 'Prenatal exposure to environmental phenols: concentrations in amniotic fluid and variability in urinary concentrations during pregnancy', *Environ Health Perspect,* 121 (10), 1225-31.
Rocha, G. H. O., et al. (2016), 'Trace metal levels in serum and urine of a population in southern Brazil', *Journal of Trace Elements in Medicine and Biology,* 35, 61-65.
Semczuk, M. and Semczuk-Sikora, A. (2001), 'New data on toxic metal intoxication (Cd, Pb, and Hg in particular) and Mg status during pregnancy', *Med Sci Monit,* 7 (2), 332-40.
Shekhar, S., et al. (2016), 'Detection of phenolic endocrine disrupting chemicals (EDCs) from maternal blood plasma and amniotic fluid in Indian population', *Gen Comp Endocrinol*.
Strain, J. J., et al. (2008), 'Associations of maternal long-chain polyunsaturated fatty acids, methyl mercury, and infant development in the Seychelles Child Development Nutrition Study', *Neurotoxicology,* 29 (5), 776-82.
Su, P. H., et al. (2015), 'Thyroid and growth hormone concentrations in 8-year-old children exposed in utero to dioxins and polychlorinated biphenyls', *J Toxicol Sci,* 40 (3), 309-19.
van der Ven, K., et al. (1992), 'Chlorinated hydrocarbon content of fetal and maternal body tissues and fluids in full term pregnant women: a comparison of Germany versus Tanzania', *Hum Reprod,* 7 Suppl 1, 95-100.
Woodruff, T. and Morello-Frosch, R. (2011), 'Communicating about chemical body burden, with Tracey Woodruff and Rachel Morello-Frosch', *Environ Health Perspect,* 119 (5).
Zeng, Q., et al. (2015), 'Urinary metal concentrations in relation to semen quality: a cross-sectional study in China', *Environ Sci Technol,* 49 (8), 5052-9.
Zhang, Y., et al. (2009), 'Phthalate levels and low birth weight: a nested case-control study of Chinese newborns', *J Pediatr,* 155 (4), 500-4.
Zheng, J., et al. (2016), 'Polychlorinated Biphenyls (PCBs) in Human Hair and Serum from E-Waste Recycling Workers in Southern China: Concentrations, Chiral Signatures, Correlations, and Source Identification', *Environ Sci Technol,* 50 (3), 1579-86.
